# PRIDASE 2024 guidelines for reporting diagnostic accuracy studies in endodontics: Explanation and elaboration

**DOI:** 10.1111/iej.14148

**Published:** 2024-09-19

**Authors:** Maria Pigg, Venkateshbabu Nagendrababu, Henry F. Duncan, Paul V. Abbott, Ashraf F. Fouad, Casper Kruse, Shanon Patel, Dan K. Rechenberg, Nandini Suresh, Yedthare Naresh Shetty, Paul M. H. Dummer

**Affiliations:** ^1^ Department of Endodontics, Faculty of Odontology Malmö University Malmö Sweden; ^2^ Department of Restorative Dentistry, College of Dental Medicine University of Sharjah Sharjah UAE; ^3^ Division of Restorative Dentistry, Dublin Dental University Hospital Trinity College Dublin Dublin Ireland; ^4^ UWA Dental School The University of Western Australia Perth Australia; ^5^ University of Alabama at Birmingham Birmingham Alabama USA; ^6^ Section of Oral Radiology, Department of Dentistry and Oral Health Aarhus University Aarhus Denmark; ^7^ Centre of Oral Health in Rare Diseases Aarhus University Hospital Aarhus Denmark; ^8^ Department of Endodontics The Faculty of Dentistry, Oral and Craniofacial Sciences at Kings' College London London UK; ^9^ Guy's & St. Thomas NHS Foundation Trust London UK; ^10^ Department of Conservative and Preventive Dentistry University of Zürich Zürich Switzerland; ^11^ Department of Conservative Dentistry and Endodontics, Faculty of Dentistry, Meenakshi Ammal Dental College and Hospital Meenakshi Academy of Higher Education and Research (MAHER) Chennai Tamilnadu India; ^12^ Department of Clinical Sciences, College of Dentistry Ajman University Ajman UAE; ^13^ Centre of Medical and Biomedical Allied Health Sciences Research, Deanship of Graduate Studies and Research Ajman University Ajman UAE; ^14^ School of Dentistry, College of Biomedical and Life Sciences Cardiff University Cardiff UK

**Keywords:** diagnostic accuracy studies, endodontics, PRIDASE 2024, reporting guidelines, root canal treatment

## Abstract

The Preferred Reporting Items for Diagnostic Accuracy Studies in Endodontics (PRIDASE) 2024 guidelines are based on the Standards for Reporting of Diagnostic Accuracy Studies (STARD) 2015 guidelines and the Clinical and Laboratory Images in Publications (CLIP) principles, with the addition of items specifically related to endodontics. The use of the PRIDASE 2024 guidelines by authors and their application by journals during the peer review process will reduce the possibility of bias and enhance the quality of future diagnostic accuracy studies. The PRIDASE 2024 guidelines consist of a checklist containing 11 domains and 66 individual items. The purpose of the current document is to provide an explanation for each item on the PRIDASE 2024 checklist, along with examples from the literature to help readers understand their importance and offer advice to those developing manuscripts. A link to the PRIDASE 2024 explanation and elaboration document is available on the Preferred Reporting Items for study Designs in Endodontology (PRIDE) website (https://pride‐endodonticguidelines.org/pridase/) and on the International Endodontic Journal website (https://onlinelibrary.wiley.com/page/journal/13652591/homepage/pride‐guidelines.htm).

## INTRODUCTION

### The need for the Preferred Reporting Items for Diagnostic Accuracy Studies in Endodontics (PRIDASE) guidelines

The Preferred Reporting Items for Diagnostic Accuracy Studies in Endodontics (PRIDASE) 2024 guidelines were developed to improve the accuracy, transparency, completeness and reproducibility of diagnostic accuracy studies within the specialty of endodontology (Nagendrababu, Abbott, et al., 2021). Similar to clinical studies in all healthcare disciplines, studies on diagnostic accuracy within endodontics are at risk of bias due to methodological deficiencies (Korevaar et al., 2015). Some of these may be difficult or even impossible to avoid, but recommendations and guidelines describing how to best *conduct* a study will improve the quality of the research by identifying those areas in which bias may occur so that scientific weaknesses can be minimized when the study is designed. As a consequence, the results of such studies will then be as robust and valid as possible, and clinical recommendations on the use of diagnostic tests based on studies of diagnostic accuracy will be as accurate as possible.

In contrast to methodological quality, the focus of the PRIDASE 2024 guidelines is on the quality of *reporting*. Thus, compliance with the PRIDASE 2024 guidelines when *reporting* diagnostic accuracy research in a scientific paper will allow readers to judge whether the results of a study are applicable to their own clinical practice. It is recognized that the performance (sensitivity and specificity) of almost any diagnostic test will vary depending on the setting, the population tested, the testing protocol (techniques, instruments, devices, laboratory tests, etc.), as well as the training and expertise of examiners who perform the test and evaluate its outcome. Therefore, the relevant details of a study must be reported clearly so that readers understand how the study was performed. From a clinical perspective, if the reporting is insufficient and/or unclear, the results are less useful as a clinical guide and may risk being misinterpreted. In a worst‐case scenario, this may lead to misguided policies and treatments and have a negative impact on the patient experience and the outcome of treatment (Cohen et al., 2016). From a research perspective, high‐quality reporting will help those who wish to replicate the study in similar or different populations. In addition, from a broader perspective, it will also allow the results of a study to be included in systematic reviews and meta‐analyses designed to assess the body of evidence for the validity of a specific diagnostic method.

### PRIDASE 2024 explanation and elaboration document

This document provides comprehensive explanations for each item within the PRIDASE 2024 checklist, with the aim of supporting researchers in the design and reporting of studies on diagnostic accuracy and providing advice for peer‐reviewers and editors when reviewing manuscripts submitted for publication. For each item in the checklist, examples of good reporting practice (i.e., reproduced extracts from diagnostic accuracy studies in endodontics, other dental/medical specialities, other study designs or hypothetical examples) are provided to further enhance understanding and inspire authors to meet high standards when preparing manuscripts of diagnostic accuracy studies in endodontics. The in‐text citations or web sites have been removed from the examples provided, and abbreviations have been expanded in their entirety to aid understanding.

#### Item 1a: Title—The Title must identify the manuscript as a diagnostic accuracy study, for example by mentioning the relevant measure(s) of accuracy (such as sensitivity, specificity, predictive values, likelihood ratios or area under the receiver operating characteristic curve [AUC‐ROC])

##### Explanation

Using the term ‘diagnostic accuracy’ or one (or more) relevant measures of accuracy in the Title clearly identifies the manuscript as a study of diagnostic accuracy, thereby facilitating its indexing in databases. Using such terms will also allow the study to be sourced more effectively by readers and during literature searches.

##### Example 1a.1

From Khademi et al. (2022)—‘In Vitro Diagnostic Accuracy and Agreement of Dental Microscope and Cone‐Beam Computed Tomography in Comparison with Microcomputed Tomography for Detection of the Second Mesiobuccal Canal of Maxillary First Molars’.

##### Example 1a.2

From Kanagasingam et al. (2017)—‘Diagnostic accuracy of periapical radiography and cone beam computed tomography in detecting apical periodontitis using histopathological findings as a reference standard’.

#### Item 1b: Title—The subject area(s) of interest must be specified in the Title, using words and phrases that clearly identify the clinical issue

##### Explanation

Brief descriptive terms and words must be used in the title to help readers identify the focus and key elements of the study. For example, such terms may relate to the nature of the diagnostic test, the target condition, the population and/or the setting of the study.

##### Example 1b.1

From Khademi et al. (2022)—‘In Vitro Diagnostic Accuracy and Agreement of Dental Microscope and Cone‐Beam Computed Tomography in Comparison with Microcomputed Tomography for Detection of the Second Mesiobuccal Canal of Maxillary First Molars’.

#### Item 2a: Keywords—The Keywords must indicate the specific area(s) of interest using MeSH terms, if available

##### Explanation

Including between two and five relevant keywords (depending on the journal) will help readers identify peer‐reviewed manuscripts in their specific area of interest. Using specific keywords in MEDLINE, also known as MeSH terms, will facilitate database indexing and improve the outcome of electronic literature searches. For example, ‘Sensitivity and Specificity’, ‘ROC Curve’, ‘Likelihood Functions’ and ‘Predictive Value of Tests’ with related entry sub‐terms such as ‘Positive Predictive Value’ and ‘Negative Predictive Value’ are MeSH terms. MeSH terms included in the Title should *not* be repeated as keywords as they are also indexed.

##### Example 2a.1

From Singh et al. (2021)—‘Cone Beam Computed Tomography, Diagnostic Accuracy, Gold Standard, Periapical Radiography, Sensitivity, Specificity’.

#### Item 3a: Abstract—The Introduction must briefly explain the background, rationale or justification for the study

##### Explanation

If the journal regulations permit, the Introduction of the Abstract must identify the relevance of the study in relation to gaps or inconsistencies in existing knowledge or where there is insufficient information on the topic. This will provide a justification for the study and indicate its significance for the clinical situation, or other diagnostic situation.

##### Example 3a. 1

From Beacham et al. (2018)—‘Limited field cone‐beam computed tomography (CBCT) imaging has become a modality frequently used by endodontists to evaluate the teeth and surrounding tissues of their patients. Accurate image interpretation is vital to obtain needed treatment information as well as to discern coincidental findings that could be present. The goal of this study was to determine the accuracy of CBCT volume interpretation when performed by endodontists and endodontic residents’.

#### Item 3b: Abstract—The Aim(s) and Objective(s) of the study must be provided

##### Explanation

The aim and objectives must be clearly described. It can be helpful to define the aim in a sentence that starts ‘The aim of this study ….’ or similar phrasing. Precision in detailing the objective(s) will enable the readers to quickly appraise whether the results are relevant to their own population or can be considered generalizable. For clarity in the study objective(s), the use of PICO elements is recommended: Patients/Population, Intervention (index test), Comparison (reference test) and Outcome (diagnostic accuracy measure(s)).

##### Example 3b.1

From Ghouth et al. (2019)—‘The aim of this study was to assess whether laser Doppler flowmetry is more accurate than the conventional pulp sensibility tests (electric pulp test and ethyl chloride) in assessing the pulpal status of permanent anterior teeth in children and to identify the laser Doppler flowmetry's Flux cut‐off threshold’.

#### Item 3c: Abstract—The Methodology must provide essential information on the study design as well as describe the reference standard and index test(s)

##### Explanation

The Methodology section within the Abstract must briefly explain how the study was performed and also provide essential information on the study design (diagnostic accuracy, test accuracy, diagnostic prediction, etc., prospective or retrospective, ex vivo), data collection, eligibility criteria for participants/samples, whether these formed a consecutive, random or convenience series; setting, location(s) and relevant dates and statistical analysis. A description of the reference standard and index test(s) must be provided. This information will help readers to quickly appraise potential sources of bias in the study design, understand the index test and the reference standard, evaluate the statistical propriety, and estimate to which degree the study results may apply to other populations with different disease prevalence and spectrums. Due to potential word limits in the Abstract section, authors have flexibility in highlighting only the most essential aspects of their study.

##### Example 3c.1

From Das and Adhikari (2021)—‘Thirty‐five patients having periapical lesions associated with anterior teeth requiring endodontic therapy were included in the study. The lesions were analyzed using IOPA radiographs, CBCT scans, and USG with color Doppler (CD). Periapical surgery ensued and enucleated tissue samples were subjected to histopathological analysis. To evaluate the accuracy, diagnoses made by each of the three modalities were compared with the gold standard histopathological reports, and the diagnostic accuracy, sensitivity, and specificity of each were calculated’.

##### Example 3c.2

From Hazard et al. (2021)—‘Data collected from 825 patients treated in the Advanced Education Program in Endodontics at the University of Iowa, USA were analysed. The experimental group included 425 teeth with FCR, whilst the control group consisted of 400 teeth with natural crowns (NC). The pulp sensibility test results, tooth type, tooth number, type of crown, age, gender, presence or absence of caries and recent use of analgesics were recorded. Bivariate analyses were performed to assess the variables associated with the accuracy of dental pulp sensibility tests for either teeth with crowns or teeth without crowns using chi‐square tests, Fisher's exact tests, Cochran‐Mantel‐Haenszel tests, and the Wilcoxon rank‐sum tests. A *p*‐value of less than .05 was used as a criterion for statistical significance, and a *p*‐value in .05 < *p* < .10 was used as a criterion for marginal relevance’.

#### Item 3d: Abstract—The Results must describe the number of subjects/specimens with and without the target condition that were included in the analysis and estimates of any accuracy measures applied and their precision

##### Explanation

Essential information includes the prevalence of the target condition in the analyses, as well as the precision of the point estimates for each reported measure of accuracy, such as 95% confidence intervals. The former information reveals the presence of missing data, and the latter information allows the readers to appraise how representative the study population is.

##### Example 3d.1

From Hazard et al. (2021)—‘The sensibility test results for full‐coverage restorations (FCR) teeth had an accuracy of 0.866; sensitivity of 0.835; specificity of 0.879; a positive predictive value of 0.746; and a negative predictive value of 0.926. The data indicated a significant difference in the accuracy of pulp sensibility test results between the experimental and control groups (*p* < .001). Although the cold test in FCR teeth still had high accuracy, teeth with natural crowns (NC) were significantly more likely to have true‐positive and true‐negative results (91.5% NC vs. 86.6% FCR, *p* = .024). No significant differences between FCR and NC were found concerning gender, tooth type, type of crown, the presence of abutment and recent use of analgesic (*p* > .05)’.

##### Example 3d.2

From Baratto‐Filho et al. (2020)—‘Nine instruments were identified in digital periapical radiography (DPR) (37.5%) and none in the cone‐beam computed tomography (CBCT) protocols (*p* > .05). The type of instrument (stainless steel hand file or reciprocating instrument) did not influence the identification of the separated instrument (*p* > .05). This study showed that DPR is the most accurate and sensitive imaging technique, with 83.3% and 37.5%, respectively’.

#### Item 3e: Abstract—The main findings of the principal aim(s) must be interpreted and summarized in the conclusion, with the clinical implications being highlighted

##### Explanation

The Conclusion within the Abstract should summarize the general interpretation of the results and explain how the results address the gap(s) in knowledge or verify/challenge existing knowledge identified in the Introduction of the Abstract. The implications for other clinical or diagnostic situations should be indicated, avoiding over‐generalization or speculation. If a part of the knowledge gap still remains in relation to the aim of the study, it should be indicated as a limitation, rather than an aim of future studies.

##### Example 3e.1

From Schloss et al. (2017)—‘CBCT analysis allowed a more precise evaluation of periapical lesions and healing of endodontic microsurgery than periapical films. Significant differences existed between the 2 methods. Over the observation period, the mean periapical lesion sizes significantly decreased in volume. Given the correct indications, the use of CBCT imaging may be a valuable tool for the evaluation of healing of endodontic surgery’.

#### Item 3f: Abstract—The source(s) of funding must be provided

##### Explanation

A brief statement describing how the study was funded (if applicable) helps readers to assess at the outset any potential conflicts of interest. The statement should include direct (money) and indirect funding (e.g., nurses and clinical facilities) from any source, including from the host clinic/institution.

##### Example 3f.1

From Jeong et al. (2023)—‘This work was supported by the BC Centre for Disease Control and the Canadian Institutes of Health Research (CIHR) [Grant # NHC‐348216, PJT‐156066, and PHE‐337680]. DJ has received Doctoral Research Award (#201910DF1‐435705‐64343) from the Canadian Institutes of Health Research (CIHR) and Doctoral fellowship from the Canadian Network on Hepatitis C (CanHepC). CanHepC is funded by a joint initiative of the Canadian Institutes of Health Research (CIHR) (NHC‐142832) and the Public Health Agency of Canada (PHAC)’.

##### Example 3f.2

From Cao et al. (2024)—‘Early administration of simnotrelvir plus ritonavir shortened the time to the resolution of symptoms among adult patients with Covid‐19, without evident safety concerns. (Funded by Jiangsu Simcere Pharmaceutical; ClinicalTrials.gov number, NCT05506176.)’.

#### Item 3g: Abstract—The name of the registry and registration number must be provided (if applicable)

##### Explanation

If the study protocol was a clinical trial related to diagnostic accuracy and was registered *a priori* in a clinical trial registry, providing the registry name and registration number will help editors and reviewers as well as readers to identify the study within the registry. Registration may not be relevant for every diagnostic accuracy study type.

##### Example 3g.1

From Chen et al. (2023) – ‘Clinical trial registration number: ChiCTR2100042312’.

#### Item 4a: Introduction—The scientific background and rationale for the study must be provided, including existing knowledge, and existing gap(s), uncertainties and inconsistencies. When information on the topic is of insufficient quality this should also be highlighted. The scientific rationale, mechanisms of action and/or principles of new diagnostic technologies should be briefly explained. The intended use and clinical role of the index test must be specified (such as screening/triage or as the basis for treatment decisions)

##### Explanation

The Introduction should review the relevant knowledge on the clinical or other diagnostic problem and identify the remaining uncertainty or gaps in knowledge, along with clinical implications. Explanations of novel technologies, algorithms or automation protocols should be described. Clarifying the intended use of the evaluated test(s) (index test[s]) in clinical or other settings and defining its/their role in diagnostics, screening, prognosis, treatment selection and/or treatment monitoring, as well as the relation to the reference test may help readers to evaluate the implications of the study results.

##### Example 4a.1

From Ramis‐Alario et al. (2021)—‘The main drawbacks of two‐dimensional diagnostic methods in their reliability rely in its limited information about size, extension and location of the periapical lesion, because of compression of three‐dimensional structures, geometric distortion and anatomic noise obscuring diagnostic clarity of the region of interest. Moreover, in the vestibular plane of periapical radiographs, the information provided is limited due to bone superimposition that makes it difficult to observe periapical radiolucent areas. In addition, the size of the periapical radiolucency may be affected by the orientation of the film and tube head. Besides, CBCT has been recommended in cases where apical surgery is being considered. No studies to date have investigated the sensitivity of the mentioned two‐dimensional radiographic techniques (periapical and panoramic) versus CBCT both before surgery and in the course of follow‐up. Likewise, no comparisons have been made of the periapical areas obtained with these two‐dimensional techniques versus the volumes obtained with CBCT before and after surgical treatment’.

##### Example 4a.2

From Torres et al. (2023)—‘Recently, a sleeveless 3D printed guide was used to treat an upper premolar. Instead of using a metal sleeve to guide the bur, the handpiece is guided by guiding rails placed against each other on the sides of the tooth. As a result, (1) vertical space is reduced, which (2) improves accessibility in posterior teeth, (3) there is direct visibility of the tooth during treatment, and (4) better water cooling. Additionally, since no sleeve is used, the (5) total cost of the guide is reduced. There is (6) no need for a dedicated bur; therefore, the procedure can be fully guided with the use of diamond burs to drill first on enamel and later longer carbide burs to further drill on dentine.

Although promising, no data is currently available concerning its accuracy. Therefore, this study aims to assess the accuracy of sleeveless guided endodontics for guided root canal treatment of severe PCO in 3D printed jaws. Additionally, a sleeveless guided endodontic treatment of a complex lateral incisor is presented to illustrate the use of the guide in a clinical situation’.

#### Item 4b: Introduction—The specific aim(s) and objective(s) of the study must be provided, including hypotheses

##### Explanation

The scientific background and identified knowledge gaps should lead to a research question and the identification of specific aim(s) and objective(s) of the study. These must be described clearly using professional language and established terminology for diagnostic research. The aim(s) and objective(s) stated in the Introduction must be the same as in the Abstract, and differences in wording between the two sections should be minimized to avoid confusion. For clarity, the use of PICO elements, or a variation of this that better matches the research question, is recommended (Luijendijk, 2021). This recommendation is further supported by Cochrane's recommended objective for reviews on diagnostic accuracy: ‘To determine the diagnostic accuracy of [index test] for detecting [target condition] in [participant description]’ (Deeks et al., 2013).

Hypotheses should also be provided. In studies of diagnostic accuracy, statistical hypothesis testing usually involves a predefined minimum level of acceptable values of, for example, sensitivity, specificity, or other measures. Other approaches can also be used such as a null hypothesis predicting no difference between tests, or a hypothesis of equality or noninferiority in accuracy when multiple index tests are compared. To avoid *post‐hoc* data dredging, hypotheses should be defined *a priori*, based on assumptions derived from pilot studies or from the results of previous studies, and used to guide sample size calculation, if applicable, with consideration of the number of groups included (Cohen et al., 2016).

##### Example 4b.1

From Mertens et al. (2021)—‘In the present randomized trial, we aimed to compare the accuracy and decision‐making impact of an AI (artificial intelligence; specifically, dentalXrai Pro) for proximal caries detection by dentists. Our hypothesis was that dentists using AI were significantly more accurate than those without AI’.

#### Item 5a: Methods—The information (name*, reference number, and date) of an ethics committee's approval, such as an Institutional Review Board, must be disclosed (if applicable)

##### Explanation

Ethical aspects must be considered in studies of diagnostic accuracy similar to other studies involving human subjects or cadavers. Authors and study staff need to maintain professional standards of care and avoid exposing the research subjects to unnecessary suffering or other risks associated with delayed treatment due to a delayed diagnosis. Authors must follow national, regional and local regulations regarding ethical review, and obtain ethical approval from an institutional review board or equivalent prior to initiating the data collection. If no ethical approval was granted/needed and an exemption obtained by the relevant ethics authority, this must be clearly described in the manuscript.

In addition to the ethical review requirements, other regulations may also apply; for example, for laboratory tests involving human tissue samples (teeth, pulp/periapical tissue, fluid, blood, saliva, etc.) biobank regulations may apply, and for radiographic studies, radiation protection regulations. Authors are responsible for obtaining all necessary permissions for involving research subjects with and without the target condition, and information about permissions should be disclosed along with the ethical approval details. Furthermore, authors should respect the right to privacy of human subjects and not publish identifying information that can be traced back to the individual (such as names or full‐face photographs).

*To maintain peer review blinding, authors should not name the institution that granted the ethical approval during the review process; the details should be added after the review process has been completed.

##### Example 5a.1

From Doğramaci et al. (2021)—‘This retrospective cross‐sectional study received ethical approval from the Human Research Ethics Committee of the University of Adelaide (H‐2018‐120)’.

##### Example 5a.2

From Ramis‐Alario et al. (2021)—‘The project is approved by the ethical committee of the University of Valencia (approval number H1523379927800)’.

##### Example 5a.3

From Jonsson Sjögren et al. (2019)—‘The Regional Ethics review board in Uppsala (daybook no.2014/197), the regional committee for ionizing radiation protection in Orebro and the regional public health service in Orbero county approved the study’.

#### Item 5b: Methods—The process used for acquiring and storing informed consent must be described

##### Explanation

For prospective studies, the authors must obtain informed consent from subjects (and/or their guardians, if applicable) to participate and (if relevant) for their tissue samples to be stored and used for research purposes. The process of acquiring consent must be transparent and the manner of storing their consent secure. For example, ‘The signed consent forms were stored in an envelope and sealed with the title and ethical clearance number written on the outside, which was locked in a file cabinet and was not accessible to anyone other than the investigators of the study’. For retrospective record‐based studies, the details on waiver of consent (if given and approved) must also be provided.

In many parts of the world, research subjects have a right to information about which data are collected, for what purpose, and how it will be processed and stored. This must be respected, and if relevant also reported. One example is the European General Data Protection Regulation (GDPR; https://eur‐lex.europa.eu/eli/reg/2016/679/oj).

##### Example 5b.1

From Singh et al. (2021)—‘The study was carried out after clearance from the Institutional ethical board (PGIDS/IEC/2016/102) and the trial was enlisted on clinicaltrials.gov (NCT04689126). All patients were informed of the purpose of the study and informed consents were obtained’.

#### Item 5c: Methods—The registration number and name of registry must be provided

##### Explanation

If the study protocol was pre‐registered in a registry for clinical trials (such as ClinicalTrials.gov or a WHO Primary Registry), the details of registration must be provided. Pre‐registration has many advantages and is strongly recommended (Bossuyt et al., 2015; Cohen et al., 2016). Registering a protocol *a priori* in a publicly accessible database is crucial because it reduces the likelihood of selective outcome reporting. It also makes it possible for fellow researchers to identify ongoing studies ahead of publication and thus prevent redundant study duplications, and to follow‐up that study results are indeed reported. It also allows reviewers and readers to identify deviations from the prospective study protocol, such as the eligibility criteria or planned analyses. Prospective registration can be regarded as a sign of quality, and providing registry information facilitates the identification of the study in the registry. It is recognized that not every study type requires pre‐registration.

##### Example 5c.1

From Chen et al. (2023)—‘The study is a case series of clinical trials conducted at one clinical center of the Department of Cariology and Endodontics at the Stomatological School and Hospital, Wuhan University; it was registered with the Chinese Clinical trial Registry (ChiCTR2100042312)**’**.

##### Example 5c.2

From Singh et al. (2021)—‘The study was carried out after clearance from the Institutional ethical board (PGIDS/IEC/2016/102) and the trial was enlisted on clinicaltrials.gov (NCT04689126)’.

#### Item 5d: Methods—Information on where the full study protocol can be accessed must be provided

##### Explanation

The full study protocol is rarely possible to include in the publication but is valuable for other researchers who may want to replicate the study or access specific details to reassess its validity, and for practitioners interested in implementing the clinical procedures. The study protocol may have been published previously in a scientific journal, posted on a website, or provided as online supplementary material to the current manuscript. The information about how to access the protocol may be given for example as a reference, a weblink or as information on whom to contact to obtain the protocol.

##### Examples 5d.1

From El Karim et al. (2023)—‘An *a priori* protocol for the COSET project was published and registered in COMET (https://comet‐initiative.org/Studies/Details/1879)’.

#### Item 5e: Methods—The timeline of the study must be included and describe whether data collection was planned before the index test and reference standard were performed (prospective study) or after (retrospective study)

##### Explanation

Prospective definition of the study question and planning of the data collection before the index test and reference standard is performed allows researchers to optimize the study protocol, for example by standardization of the test procedure and blinding of examiners to the index test/reference standard results. If the study is planned when patients have already undergone testing, the data are usually collected by reviewing patient records or from a registry. This may induce selection and recall bias since some eligible individuals may be missed, and some data points may be missing or ambiguous, possibly resulting in an overall lower quality of data. Although it is often more convenient, and indeed also sometimes preferable, to use existing data to plan and execute a prospective study, it is important that the timing details are transparent so that the readers may assess which types of, and to what degree, bias may be inherent in the design.

##### Example 5e.1

From Kielbassa et al. (2003)—‘The root canal length was clinically determined with the electronic apex locator Root ZX (Morita, Tokyo, Japan). The device was operated according to the manufacturer's instructions; contamination of the teeth's access chambers with saliva or blood was avoided, and dryness was assured during measurements by means of cotton rolls. Reference points were marked on the tooth crown with a felt pen to facilitate accurate reinsertion of the files. The reference points, the number of canals, and whether bleeding was noted (vital or necrotic pulp) were recorded.

After careful extraction of the teeth, a “real” length was determined using the same files and points of reference’.

##### Example 5e.2

From Zhang et al. (2019)—‘CBCT images of 29 endodontically treated teeth from 29 patients were analyzed. Patients included those referred to our institution from September 2014 to September 2018 for definite diagnosis and treatment, including 9 men and 20 women (average age 55.2 [range: 22–77] years)’.

#### Item 5f: Methods—The important features of the study design, including measures of diagnostic accuracy (such as sensitivity, specificity, predictive values, likelihood ratios, or AUC‐ROC), must be provided in the Methods section

##### Explanation

To ensure readers are able to fully understand the study, a comprehensive description of the study protocol including the definition of the outcome measures of accuracy must be provided in the Methods section.

##### Example 5f.1

From El Sayed and Gaballah (2021)—‘The numbers of patients who responded to the post anesthetic cold test with true positive (TP), false positive (FP), true negative (TN), and false negative (FN) responses were calculated and compared to the results of the gold standard test. Table summarizes the meanings of these parameters. Using MedCalc's free online “diagnostic test statistical calculator,” sensitivity (SN), specificity (SP), positive predictive value (PPV), negative predictive value (NPV), and accuracy (AC) with confidence intervals (95% CI) were calculated for the cold test. Youden index was determined for the overall diagnostic precision of the post anesthetic cold test using the following equation: specificity + sensitivity − 1. Finally, for the cold test, a receiver operating characteristic (ROC) curve analysis was used to measure overall predictive power and quantify the region under the curve (AUC)’.

##### Example 5f.2

From Singh et al. (2021)—‘Accuracy was assessed as sensitivity and specificity in comparison to the reference gold standard. Difference in diagnostic sensitivity and specificity between the imaging modalities was analysed by McNemar's test with 5% significance level. To determine whether the differences in the findings between gold standard and imaging techniques were attributable to demographic data (patient's age, gender, location [maxilla vs. mandible], tooth type [anterior vs. posterior] and status of root canal treatment), a logistic regression model was applied’.

#### Item 5g: Methods—The rationale for, and method of, the sample size calculation, preferably with reference to a pilot study, or based on data from the published literature, must be included with added detail as to why the defined sample size makes the study worthwhile

##### Explanation

The optimum sample size depends on the specific objectives and hypotheses of the study. To determine the necessary number of subjects (or specimens), assumptions about expected outcomes need to be made. These assumptions must be transparent and should be in line with previous findings as reported in the literature (if available) or a pilot study. The rationale for the intended sample size must be described.

Reporting the method or procedure for sample size determination allows the reader to judge whether the assumptions made are appropriate in relation to the clinical setting and existing scientific evidence. Reporting the intended sample size also makes it clear whether the study reaches the intended level of precision for the accuracy estimates.

In general, small sample sizes, and/or large numbers of experimental groups, will result in low precision that is often revealed by large variability, for example, wide 95% confidence intervals surrounding the point estimate. Determined prospective criteria for a minimum acceptable precision of the results and performing an *a priori* sample size calculation based on this may therefore improve the study. If such criteria were applied, they should be described. In addition, information must be provided on whether the targeted number of subjects (or specimens) was reached since this affects the ability of the study to answer the research question with sufficient certainty. When no sample size calculation is performed, the reasons must be provided.

##### Example 5g.1

From Singh et al. (2021)—‘The sample size was estimated using data from a previous study, which found that the detection rates of periapical lesion to be 31.5% with PR and 52.2% with CBCT. With 80% power and an alpha error at 5%, the minimum sample size was computed to be 83 for detecting the differences in detection rate between CBCT and PR. Each tooth was considered as a single unit’.

##### Example 5g.2

From El Sayed and Gaballah (2021)—‘The sample size was determined based on the statistical formula stated by Chavarría‐Bolaños et al. The sample size calculation was performed with a type I error of 0.05 (significance of 95%) and statistical power of 80%. *P* is the average of sensitivity percentage values of the cold test (0.84) and gold standard (1.0) as reported in a previous study. *P*1 is the percentage value of sensitivity of the gold standard in the previous study. *P*2 is the percentage value of sensitivity of the cold test in the same previous study.

After applying exclusion criteria and based on the previous formula, the required sample size was 44 patients however, fifty‐seven patients were included in the current study. This number of patients was deemed sufficient to demonstrate any differences that could be attributed to the diagnostic tests used’.

#### Item 5h: Methods—The inclusion and exclusion criteria, as well as the sources and methods of participant/sample selection, must be described

##### Explanation

The composition of the participants has the potential to have a major effect on the study outcomes. Therefore, inclusion and exclusion criteria for study participation must be clearly and comprehensively described.

The source and method of participant selection must also be described (e.g., if the participants are recruited from the same or different clinics or centres, and how they are identified), and in the case of a retrospective study, the source of data (e.g., patient charts or another registry) and the method to identify eligible participants (e.g., the search of a database). Source, as well as the method of selection, may influence the disease spectrum and prevalence of the target condition as well as the presence of any alternative conditions. The diagnostic accuracy is affected by these factors, which must be transparent to allow readers to assess the relevance of the results for their population and the intended use of the test.

It must also be transparent If the same eligibility criteria are used for subjects with and without the target condition (cohort study or single‐gate), or if the criteria are different between the two (or more) groups (case–control study or multiple‐gate). Secondary criteria of exclusion, for example, motivated by unfeasibility or safety concerns, must also be described.

##### Example 5h.1

From El Sayed and Gaballah (2021)—‘The inclusion criteria to enroll in the study were as follows: patients must have symptoms of symptomatic irreversible pulpitis in the first mandibular molar due to caries or defective direct restoration. The patients should have a recent history of acute spontaneous pain categorized as moderate (scale 4–6) to severe (scale 7–10) based on the 0–10 Numerical Rating Scale (NRS). This scale was used to eliminate any bias caused by varying levels of pain during selection. The exclusion criteria were as follows: pregnancy (female patients), gross caries rendering the tooth unrestorable, teeth with artificial crowns, advanced periodontitis, radiological evidence of root resorption, teeth with narrow pulp chambers, cracked teeth, patients with a history of significant adverse reaction to local anesthetics including the allergy, uncontrolled diabetes or hypertension, intake of drugs that may interfere with sensation in the orofacial region, severe dental/needle phobia and inability to give informed consent. Moreover, the study did not include patients with delayed or lack of response to cold testing, the presence of extensive periapical pathologies, or necrotic coronal pulp tissue that occurred whilst the pulp chamber was being penetrated’.

#### Item 5i: Methods—The criteria used to identify potentially eligible participants (such as symptoms, preoperative status, results from previous tests and inclusion in the registry) must be described (if applicable)

##### Explanation

Eligibility criteria are related to the nature and stage of the target condition, and the disease spectrum of the sample must be transparent. If potentially eligible participants are identified by specific clinical findings or symptoms, results on previous testing, or perhaps by their inclusion in a database, this must be described. The intended future use of the index test should be matched to the study sample and setting (if relevant), for example, if the evaluated test is intended for identifying pulp necrosis in painful teeth, then all the study participants should have symptoms, and vice versa: if the test is intended specifically for the identification of active apical disease associated with asymptomatic root filled teeth, the study participants in the group with the target condition should be patients without symptoms from their root filled tooth, and nonroot filled teeth should not be included.

##### Example 5i.1

From Hazard et al. (2021)—‘The presence or absence of bleeding in the pulp chamber was recorded by direct observation using the dental operating microscope. Detection of bleeding in both the pulp chamber and coronal aspect of the root canal/s was necessary to make a diagnosis of a vital pulp. In the absence of vital tissue within the pulp chamber, the pulp was considered necrotic. When bleeding tissue was not observed in all of the canals of a multi‐rooted tooth, the status was classified as necrotic. In these cases, the number of the bleeding canal(s) and type were recorded’.

#### Item 5j: Methods—Details on whether the participants constituted a sequential, random, community‐based or convenience series must be provided, if applicable

##### Explanation

This issue is relevant for the generalizability of the results. For readers to be able to assess the risk of selection bias, the details of the sample must be transparent. A consecutive series of patients fulfilling the inclusion criteria may be representative of the intended target population to a greater extent than a subsample, but that depends on the selection of the latter. Whilst a random selection from all eligible patients in the study location is comparable to a consecutive series, a nonrandom selection may not be. For example, a nonwarranted restraint in inclusion based on age, gender, time or other factors may limit the generalizability to the population for which the test is intended. A convenience sample is based on the subjects being accessible to the researchers (e.g., patients attending a dental school) and may have the same flaw, if the source from which data are retrieved has limitations, for example, in the disease spectrum. For example, patients attending an emergency clinic are likely to have more severe endodontic disease, or at least more severe symptoms, than patients identified in a recall visit to their general dentist. A community‐based sample may have similar limitations if the community is not representative of the targeted population, for example concerning age distribution or disease prevalence.

##### Example 5j.1

From Vanitha and Sherwood (2019)—‘Sixty patients (36 males and 24 females) between the ages of 13 and 60 years, who were referred to Department of Endodontics for root canal treatment in mandibular first molar teeth during October 2017 and January 2018, participated in the study’.

##### Example 5j.2

From Zhang et al. (2023)—‘In a randomized double‐blind, sham feedback‐controlled design, 33 participants (16 males) were randomly assigned to the NF group receiving NF from the LAI and 33 participants (16 males) were assigned to the control group receiving sham NF from a control region of the middle temporal gyrus (MTG; see *Definition of the sham control region for the control group*). Nine participants were excluded due to not completing the whole experiment (3 participants), excessive head movement (3 participants), failure of feeling heartbeat during the heartbeat counting task (HCT) and quitted the study (1 participant), or technical problems during NF training (2 participants)’.

#### Item 5k: Methods—The setting, location(s) and date(s) of data collection must be specified

##### Explanation

The performance of the index test is influenced by the setting, for example, if performed in general or specialist clinics, which have differences in the patient population in terms of prevalence and spectrum of the target condition as well as the range and prevalence of other relevant conditions. This affects the generalizability of the results, and authors must therefore specify the setting and provide the location where the test was performed, such as the name(s) of the clinic(s), city and country. Although describing the location may compromise anonymity, such details can be masked during the submission process. In addition, the start and end dates of the patient recruitment must be given, or for retrospective studies, the time period when the index test and reference standard were performed. The rationale for this is that test procedures and disease prevalence as well as other relevant factors can change over time.

##### Example 5k.1

From Virdee et al. (2023)—‘A cross‐sectional study was conducted on the undergraduate endodontic specialty teaching clinics at Birmingham Dental Hospital between September 2021 and June 2022’.

##### Example 5k.2

From Hazard et al. (2021)—‘Data were collected from patients seen by the faculty and postgraduate students in the Endodontic clinic at the College of Dentistry between September of 2018 and June of 2019’.

#### Item 5l: Methods—Sources of data and details of methods of assessment (measurement) for each variable of interest must be provided. Comparability of assessment methods if there is more than one group must be provided

##### Explanation

The study protocol must be described in sufficient detail to allow replication of the study. Descriptions of the sample should be provided in detail for the readers to be able to interpret. For example, in a study evaluating a pulp sensibility test, it is not enough to state that the participants had ‘deep caries’ in the tooth on which the test was performed. It should also be reported which method was used to identify caries, such as clinical visual inspection, or radiographic depth of caries in dentine or distance from the pulp. If more than one group was included and the method of assessment differed between the groups, this must also be disclosed, and an appraisal of the comparability provided.

##### Example 5l.1

From Hazard et al. (2021)—‘Teeth were isolated and dried with either 2X2 gauze or air spray before testing. Subjects were directed to raise their hand at the moment of thermal sensation. A size 2 cotton pellet was sprayed with Endo Ice (1,1,1,2‐tetrafluoroethane; Hygenic Endo‐Ice Green; Coltene Whaledent, Cuyahoga Falls, OH, USA) and applied to the buccal surface of the tested teeth. The frozen cotton pellet was held on the tooth's surface for 15 s or until the subject raised their hand. The adjacent and contralateral teeth were tested, before the studied teeth, as controls for a comparative baseline response. Previously, root filled adjacent teeth (*n* = 20 teeth) were used as negative controls.

All 825 subjects included in the study were referred by their dentists for root canal treatment with a diagnosis of either symptomatic irreversible pulpitis or pulp necrosis with symptomatic apical periodontitis. They complained of spontaneous and severe pain on the tested teeth, and the need for root canal treatment was confirmed at the screening and consultation visits. Their ages range between 18 and 88 years. The natural crown group included partially restored or carious teeth. Four hundred subjects without full‐coverage restorations and 425 subjects with full‐coverage restorations participated’.

#### Item 5 m: Methods—The Index test(s) (e.g., CBCT, cold test) must be presented in sufficient detail (including techniques, equipment, software, vendors and reagents, if applicable) as should the justification for the reference standard, to ensure the study can be replicated

##### Explanation

The performance of a diagnostic test can vary. For example, a test designed to identify pulp necrosis by testing the reaction of the pulp's sensory nerves to a cold stimulus may vary regarding the exact site of application on the tooth, the temperature and physical properties of the stimulus, the preparation of the test site (isolation, dryness, etc.), the time of duration of the application and so on. The equipment may also vary, including the type of cold stimuli, the manufacturer and so on. Likewise, the medical history of the patients and any medications that may influence the results of the index test should be noted or mentioned as exclusion criteria. Any variation in the performance of the index test is a potential source of variation in diagnostic accuracy. Transparent and detailed reporting of the index test and how it is executed allows the readers to assess whether the test is feasible in their own setting and with their available equipment, and whether the study results are applicable in a certain clinical situation. This is of particular relevance for CBCT equipment which may vary significantly between dedicated and hybrid versions.

##### Example 5m.1

From El Sayed and Gaballah (2021)—‘After 15 min, the cold sensibility test was performed to identify the presence of pulpal anesthetic failure, and the results were compared to the gold standard test. The gold standard benchmark for anesthetic failure was identified as any painful sensation or discomfort during the access cavity preparation and the pulpal tissue manipulation. The level of pulp removal was standardized until a full pulpectomy was accomplished. The target tooth was fully isolated using a rubber dam and then subjected to the cold test as stated previously after the soft tissue anesthesia was verified. After a one‐minute interval, the test was repeated two more times. Regardless of the level of pain, the painful response or any discomfort of patients at any level of pulpectomy procedures was registered as a positive response’.

#### Item 5n: Methods—Information on who performed the Index test(s), and their experience and/or calibration on performing the test, must be provided

##### Explanation

The number, training, experience and calibration of the individuals performing the index test may affect the results. Inter‐examiner variability can be reduced using fewer examiners who are trained and have experience in executing the index test and interpreting its results.

##### Example 5n.1

From Kruse et al. (2017)—‘Three experienced observers (two endodontists [CK, LLK] and one oral radiologist [RSN]) evaluated all radiographic images and CBCT volumes. For both radiographic modalities, the periapical scores were registered by each observer, and a consensus was reached by selecting the most frequent score. In case of disagreement (three different scores), the three observers discussed the case until consensus was reached’.

#### Item 5o: Methods—The Reference standard must be presented in sufficient detail to identify the exact scope of the study, and for replication to be possible

##### Explanation

Similar to the index test, the performance of the reference standard may also be affected by procedural variations. The selection and description of the reference standard must be presented in sufficient detail to allow replication of the study, and to enable readers to appraise whether the reference standard is adequate. This includes, for example, the procedure of measurement; instruments, clinical electronic or imaging devices and other equipment; laboratory tests and test analyses. If the reference standard is not the same between the study groups (or it was measured differently) which groups received which reference standard must be described in detail. For example, in a study on pulp sensibility testing, the reference standard may involve direct inspection of the pulp upon initiation of root canal treatment in the group with the target condition (irreversible pulpitis or pulp necrosis), but in the group presumably without the target condition (true negatives), that is, with pulps that react to testing, within the normal range, unless root canal treatment or tooth extraction is planned for other reasons, the pulp chamber will not be opened and the state of the pulp may have to be assumed based on the absence of disease signs and/or another pulp test. In this case, different reference standards are thus applied in the two groups, and this must be adequately described. The accuracy in this case is for only cases that received root canal treatment or in which the pulp was directly inspected, and not for all cases presenting for treatment. Likewise, for a study that uses CBCT as a reference standard, to avoid confusion the same machine, or at least the same field of view, resolution and exposure characteristics must be used for all cases and detailed.

##### Example 5o.1

From Kruse et al. (2017)—‘All patients accepting surgical endodontic retreatment were re‐operated (SER‐R) by one endodontist (CK). Under local anesthesia a mucoperiostial flap was raised. Bone removal over the lesion was performed with a round bur to expose the periapical lesion. The soft tissue of the periapical lesion was carefully removed in toto by surgical spoons and immediately fixed in neutral buffered 10% formalin (pH 7.2, 20°C) for histopathological examination. The resection surface was smoothened using a fissure bur and the retrograde preparation was made with retro‐tips in an ultrasonic hand piece (Satelec Newtron P5, Satelec Acteon, Merignac, France). After hemostasis was obtained, the retro‐preparation was cleaned using 3% H_2_O_2_, dried by paper points, and filled with Mineral Trioxide Aggregate (ProRoot MTA White, Dentsply Tulsa Dental, Tulsa, OK). In one case, in which ultrasonic preparation was impossible due to a long cast metal post, the resection surface was shallowed lightly with a round diamond bur and Retroplast (Retroplast Trading, Rørvig, Denmark) used as retrograde filling material. The surgical area was thoroughly cleaned with sterile saline and the soft tissue closed using 5‐0 sutures (Vicryl, Ethicon, Somerville, NJ). Sutures were removed after 7–10 days.

The formalin‐fixed tissues were dehydrated in graded alcohols and embedded in paraffin. Sections of 3–4 μm were cut using a microtome (Leica GmbH, Nussloch Eisfeld, Nussloch, Germany) and stained with haematoxylin and eosin for routine histopathological examination under light microscopy. One oral pathologist (JR) assessed all biopsies and made standard histopathological examinations and reports’.

#### Item 5p: Methods—Information on who assessed the reference standard(s), including their experience and any calibration, must be provided

##### Explanation

Similar to the index test, the number, training, experience and calibration of the individuals performing the reference standard may affect the results. If the reference standard is complex and includes several domains or there are multiple reference standards, information on who performed the standard must be provided for each of the domains or standards, as well as their training and any calibration performed. As for index test assessment, few and adequately trained examiners with expertise and experience in the reference standard can reduce inter‐examiner variability.

##### Example 5p.1

From Le et al. (2011)—‘The observers were two final‐year postgraduate endodontic students, one with 6 years of experience since graduation from dental school and the other with 20 years' experience. A third observer was a final year undergraduate dental student undertaking an Honours degree research project.

The reliability of agreement amongst the observers was analysed utilizing free‐marginal multi‐rater kappa tests’.

#### Item 5q: Methods—The rationale for selecting the reference standard must be described

##### Explanation

The purpose of the reference standard is to establish the presence or absence of the target condition. Several different standards with this ability may be available, and the authors must provide a rationale for their choice, which may be guided by practical availability or ethical reasons, or by clinical relevance associated with the stage of the target disease (such as the degree of pulp inflammation or the level of infection). The rationale will also define the limitations of the results of the study.

##### Example 5q.1

From Kruse et al. (2017)—‘Histopathological examination as a reference standard to assess treatment outcome is often difficult to obtain as this includes a surgical intervention. In 1972, Andreasen & Rud demonstrated a correlation between findings in periapical radiographs and inflammatory status in teeth having undergone SER. No studies have been performed to examine whether this correlation also exists between findings in CBCT and histopathology’.

#### Item 5r: Methods—The definition of and rationale for test positivity cut‐offs or result categories of the index test(s) and the reference standard must be described, distinguishing pre‐specified from exploratory

##### Explanation

Index test results can be dichotomous by nature (definite positive vs. negative), categorical (e.g., low, intermediate or high degree/risk) or continuous on an interval or ratio scale. In the two latter cases, reclassification is often made, defining a cut‐off (threshold) to determine if the test result is positive (supra‐threshold) or negative (sub‐threshold). Alternatively, a ROC curve can describe the sensitivity and specificity for all possible cut‐offs. To determine the applicability of the thresholds or categories in clinical practice, how the thresholds were determined must be described. Especially relevant is whether they were defined prior to the study (pre‐specified) or after data collection (exploratory). Pre‐specified thresholds can be based on, for example, previous studies, cut‐offs used in clinical practice, or recommendations in guidelines for clinical practice or by the device manufacturer. With no pre‐specified thresholds, an exploratory approach is often taken and if the test performance is maximized in the analyses, there is a risk of over‐estimating the test accuracy. Therefore, the cut‐off definitions and the rationale for this must be clearly described.

The clinical relevance of the reference standard positivity cut‐off determines its applicability in a clinical situation. Similar to what is described above for the index test, an exploratory approach to reference standard positivity thresholds when the reference standard is not dichotomous by nature risks overestimating the accuracy of the index test. Therefore, the cut‐off definitions and rationale must be described clearly.

##### Example 5r.1

From Kruse et al. (2019)—‘For the CBCT assessments of AP, a consensus observer score for each plane (sagittal and coronal) was made by selecting the most frequent score. In case of three different scores, a mathematical consensus (the middle score) was chosen. The inter‐observer agreement was evaluated by calculating Kappa‐values for agreement amongst the observers. Radiographic consensus scores for the sagittal and coronal plane of each root were combined to one score using cross tables. The different colouring of the entries in Tables 4, 5 and 6 show the combination of the sagittal and coronal consensus scores stratified on the histopathological periapical diagnosis. CBCT scores “Periapical destruction of bone definitely not present” and “Periapical destruction of bone probably not present” were categorized “Healthy”. Scores of “Unsure” were only used according to their histopathological diagnoses when calculating the diagnostic accuracy parameters. “Periapical destruction of bone definitely present” and “Periapical destruction of bone probably present” were categorized “Diseased”’.

#### Item 5s: Methods—Whether clinical information and reference standard results were available to the performers/readers of the index test must be described

##### Explanation

When performing an index test or interpreting the test result, additional information may influence the procedure or the interpretation. This may lead to a high but biased agreement between the index test and reference standard. In some cases, this is not entirely inappropriate, since some tests require additional information for correct interpretation, which is how the index test will be used in clinical practice. Therefore, the test performance in the study may mirror its performance in practice only when additional information is available. However, the additional information may also lead to a wrong interpretation, for example, if associations between symptoms and health status are incorrectly assumed. Independent of the case, information on to what extent the reference standard results were unavailable (i.e., ‘blinded’ or ‘masked’) to the examiner or test interpreter must be transparent so that the reader is able to judge whether the reported test performance may be biased or not. A flow chart or similar describing the timing and order of application of the index test and reference standard also clarifies if the reference standard was available or not. It has to be considered that in certain situations, reference standards may not be available at the time of index testing such as histopathological assessment in a clinical study. The authors may not report this since the information is redundant.

##### Example 5s.1

From Salgar et al. (2017)—‘The endodontic diagnostic tests were performed by a single researcher who was blinded to clinical signs and symptoms, dental histories, and radiographic findings’.

#### Item 5t: Methods—Whether clinical information and index test results were available to the assessors of the reference standard must be described

##### Explanation

Similar to that described for the index test, the performance or interpretation of the reference standard may be influenced by additional information, if available. In particular, if the reference standard assessment requires subjective interpretation it may be guided by the index test result if known, which will lead to an over‐estimate of the accuracy of the index test (Whiting et al., 2013). For transparency, it must be reported if the reference standard was assessed in an operator‐masked manner, (i.e., with or without information on the index test result) and/or additional information, and whether this was consistently done for all cases. A flow chart or similar describing the timing and order of application of the index test and reference standard also clarifies if the index test result was available or not.

##### Example 5t.1

From Singh et al. (2021)—‘Radiographic images were evaluated concurrently by three blinded observers (an oral and maxillofacial radiologist [AG] and two endodontists [RY, JD]) well experienced in radiographic analysis. One case from each category were utilized for calibration preceding the image analysis which were subsequently excluded from the study (nine teeth from 7 patients). In first session, the clinical history and examination along with periapical radiographs were analysed by each observer individually. At the second evaluation, in addition to clinical data and periapical radiographic findings, the same observers were provided with CBCT images of the patients in a randomized order at the interval of at least 2 weeks’.

#### Item 5u: Methods—All statistical procedures employed in the study, including those used to account for confounding factors and in data analysis, must be described

##### Explanation

The statistical methods, including statistical tests and software used and steps taken to validate the accuracy of the data, must be described in sufficient detail for the reader to judge if the procedures were appropriate and the results reliable.

##### Example 5u.1

From Hazard et al. (2021)—‘Bivariate analyses were performed to assess the variables associated with the accuracy of dental pulp sensibility tests for either tooth with crowns or teeth without crowns using chi‐square tests, Fisher's exact tests, Cochran–Mantel–Haenszel tests and the Wilcoxon rank‐sum tests. A *p*‐value of less than .05 was used as a criterion for statistical significance, and a *p*‐value in .05 < *p* < .10 was used as a criterion for marginal relevance. Statistical analyses were performed using the statistical package SAS_ System version 9.4 (SAS Institute Inc., Cary, NC, USA)’.

#### Item 5v: Methods—Methods for estimating or comparing measures of diagnostic accuracy must be described, including assessment of internal reliability (comparison of accuracy amongst operators), if applicable

##### Explanation

The performance of a diagnostic test can be measured in multiple ways. Authors must report and justify which measures were considered appropriate based on the study objectives or hypotheses, and which methods were used to calculate the estimates. If several operators/examiners contributed, the overall and individual accuracy estimates must be described to control for inconsistency in assessments.

If a pre‐specified level of ‘acceptable diagnostic accuracy’ was defined to allow hypothesis testing in a single‐test evaluation (e.g., ‘sensitivity of at least 85% and specificity of at least 90%’) this must be described.

If two or more index tests were compared, the assumption of superiority or noninferiority of tests used in the statistical hypothesis testing must be described, and the measure of comparison specified (e.g., relative sensitivity, gain in sensitivity or relative diagnostic OR) (Hayen et al., 2010). The threshold of significance (p‐value) must be provided.

##### Example 5v.1

From Singh et al. (2021)—‘Accuracy was assessed as sensitivity and specificity in comparison to the reference gold standard. Difference in diagnostic sensitivity and specificity between the imaging modalities was analysed by McNemar's test with 5% significance level. To determine whether the differences in the findings between gold standard and imaging techniques were attributable to demographic data (patient's age, gender, location [maxilla vs mandible], tooth type [anterior vs posterior] and status of root canal treatment), a logistic regression model was applied’.

#### Item 5w: Methods—How uncertain or ambiguous index tests or reference standard results were handled must be described, if applicable

##### Explanation

Uncertain or ambiguous test/standard results refer to indeterminate or outlier results that are not clearly identifiable as either positive or negative or may have had an unidentifiable error in measurement. This can occur due to several reasons, such as technical (device) failure, insufficient sample (e.g., biopsy material), failure to complete the testing procedure, or missing information in one domain of a complex reference standard requiring information from several domains. How uncertain or ambiguous index test results or reference standard results are handled in the analysis has the potential to affect the report of test accuracy and must therefore be accounted for. There are several possible approaches, including ignoring indeterminate results altogether (e.g., with very few occurrences), reporting the frequency but excluding them from analysis, reporting them as a separate category, reclassifying all indeterminate results as false (false negatives in participants who are cases according to the reference standard and false positives in noncases; a so‐called ‘worst‐case scenario’) or as true (true positives in cases and true negatives in noncases; a ‘best‐case scenario’).

##### Example 5w.1

From Virdee et al. (2023)—‘The exact parameters including brand, insertion depth relative to the zero‐reading, ISO size and sampling duration are outlined in the ‘volume absorbance’ subheading of the results section. If any of the three cones became contaminated by profuse apical bleeding, the entire sample was abandoned’.

#### Item 5x: Methods—How missing data on the index test and reference standard were handled must be described, if applicable

##### Explanation

The reporting of how missing data was handled allows the reader to assess if and how bias due to missing data may be present in the reported diagnostic accuracy. Missing data can be handled in several different ways. Exclusion of participants without either an index test result or a reference standard result (‘complete case’ analysis) may introduce bias if missing data are more common in participants either with or without the target condition and could also lead to loss of precision. Imputation of a missing index test result with data are an alternative to exclusion, and consideration of different scenarios is one possibility. In such scenarios, the missing results are assumed to be either false (false negatives in cases according to the reference standard and false positives in noncases; a so‐called ‘worst‐case scenario’) or true (true positives in cases and true negatives in noncases; a ‘best‐case scenario’). A combination of strategies can also be applied, for example, exclusion of the participants supplemented with a ‘worst‐case scenario’ analysis.

##### Example 5x.1

From Fransson et al. (2021)—‘The SSIA database for 2009 contained registrations for 248 299 root fillings in 217 047 individuals. Per individual, we included only the first root filled tooth in our analyses. As some individuals had missing data and were excluded, the data were based on 216 764 individuals and the logistic regression on 215 940 individuals’.

##### Example 5x.2

From Dastmalchi et al. (2012)—‘After completion of the access cavity, the vitality status of the teeth was recorded by a direct visual inspection, which was considered to be the gold standard in this study. If there was any doubt about the vitality status of the tooth, that tooth was excluded from the study’.

#### Item 5y: Methods—Any analyses of variability in diagnostic accuracy must be described, distinguishing pre‐specified from exploratory

##### Explanation

Patient characteristics (including the stage of disease, symptoms, etc.), the study setting (centre), and examiners' training and experience are amongst the factors that may influence the occurrence and proportion of false results on the index test. An analysis of the variability across subgroups can reveal sources of variability that may or may not exist in the intended clinical application of the test and will help the reader understand if the reported diagnostic accuracy is generalizable to their clinical practice. An analysis of variability with pre‐specified subgroups may have less risk of bias than if determined *post hoc* (exploratory; Sun et al., 2014), and the type of variability analysis must therefore be described. An inter‐examiner agreement analysis is recommended (if relevant).

##### Example 5y.1

From Singh et al. (2021)—‘To determine whether the differences in the findings between gold standard and imaging techniques were attributable to demographic data (patient's age, gender, location [maxilla vs mandible], tooth type [anterior vs posterior] and status of root canal treatment), a logistic regression model was applied’.

##### Example 5y.2

From Pigg et al. (2016)—‘The influence of patient‐, tooth‐, and dentist‐related characteristics on the measures of validity was also analysed.

The Pearson chi‐square test analysed differences for categoric variables, and the Student *t* test was used for continuous variables. Statistical significance was assessed at *p* ≤ .05. SN and SP with 95% confidence intervals (CIs) were computed overall and for subgroups. Significant differences in SN and SP between groups were defined as nonoverlapping 95% CIs’.

#### Item 6a: Results—The number of participants/specimens who underwent the index test(s) and reference test and were included in the analyses must be described

##### Explanation

The final number of participants or specimens that underwent the index test and reference standard with conclusive results must be clearly described. A flowchart allows easy understanding of the flow of participants/specimens throughout the study, from the assessment of eligibility to the number of participants/specimens with and without the target condition that had positive and negative index test results. Authors are recommended to use the STARD 2015 flowchart template (https://www.equator‐network.org/reporting‐guidelines/stard/). The flowchart should clearly show the number of indeterminate index test or reference standard results, missing data and other deviations from the planned protocol which may affect the estimate or precision of the reported accuracy.

##### Example 6a.1

From El Sayed and Gaballah (2021)—‘The final research sample consisted of 54 adult participants, 35 of whom were males (65%) and 19 of whom were females (35%). The participants' ages ranged from 18 to 40, with an average of 32.3 ± 5.5 years’.

##### Example 6a.2

**FIGURE 1:**
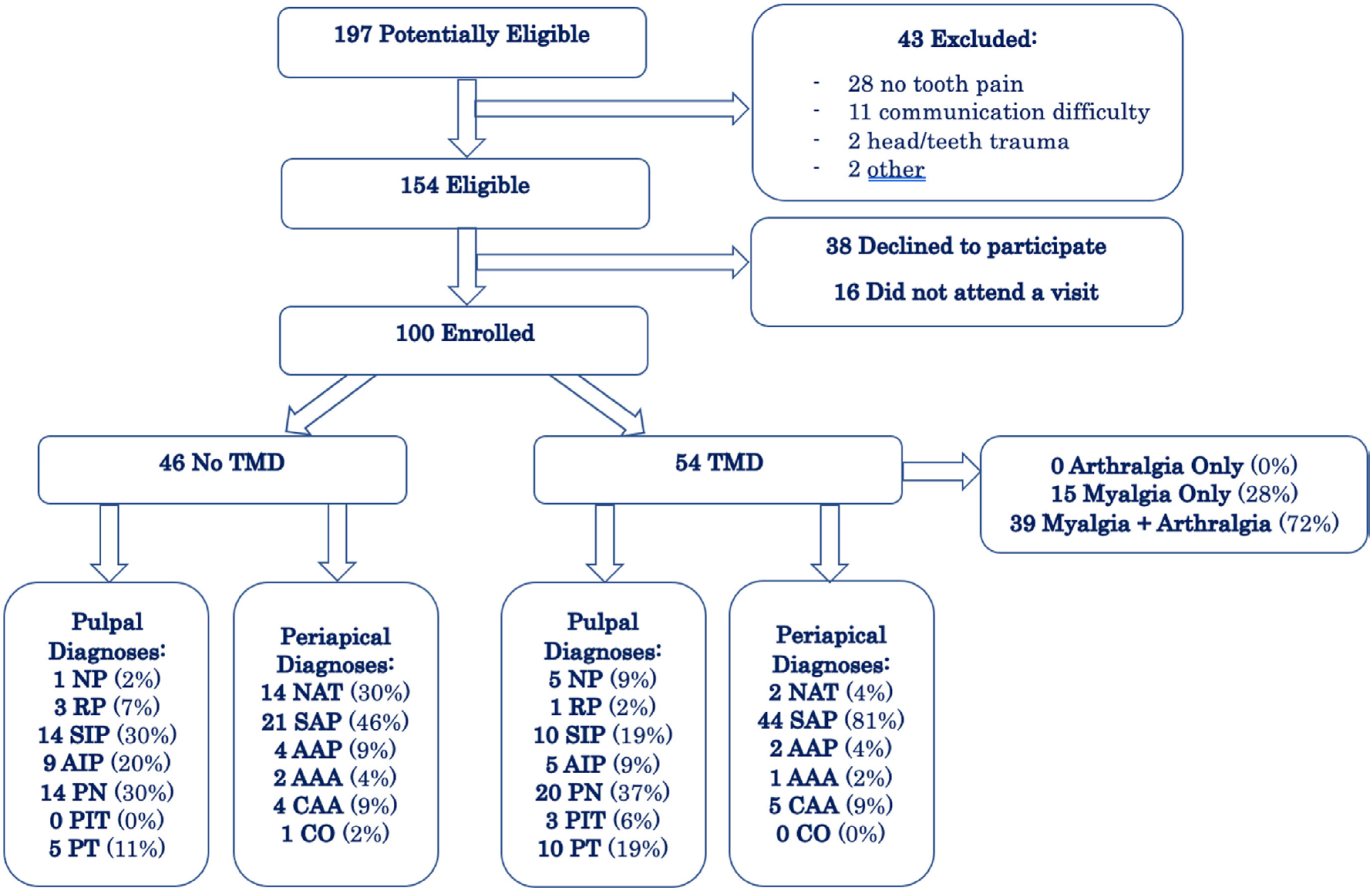
Item 6d – Flow of participants. AAA, acute apical abscess; AAP, asymptomatic apical periodontitis; AIP, asymptomatic irreversible pulpitis; CAA, chronic apical abscess; CO, condensing osteitis; NAT, normal apical tissues; NP, normal pulp; PIT, previously initiated treatment; PN, pulp necrosis; PT, previously treated; RP, reversible pulpitis; SAP, symptomatic apical periodontitis; SIP, symptomatic irreversible pulpitis; TMD, temporomandibular disorders. Reprinted from *Journal of Endodontics*, Vol 50, Daline, I. H., Slade, G. D., Fouad, A. F., Nixdorf, D. R., & Tchivileva, I. E. (2024). Diagnostic Accuracy of a Temporomandibular Disorder Pain Screener in Patients Seeking Endodontic Treatment for Tooth Pain, Pages No. 55–63, Copyright (2024) with permission from Elsevier.

From Pigg et al. (2016)—‘Figure 1. A flowchart describing the number of patients and teeth receiving index and reference tests and the frequencies of test results’.

#### Item 6b: Results—The baseline demographic and clinical characteristics of study participants must be provided, if applicable

##### Explanation

The diagnostic accuracy of a test may vary depending on several factors, including the clinical characteristics and demographic distribution of the test population. For example, in a study examining the diagnostic accuracy of a particular method to identify pulp necrosis, its accuracy may be completely different if the study population consists of adolescents suffering from dental trauma or geriatric patients with deep caries lesions. A clear description helps the reader judge if the study question is properly addressed, and whether the study findings are relevant and applicable in each given clinical situation.

##### Example 6b.1

From Virdee et al. (2023)—‘Fifty‐two PTF samples (NAT: 19; AAP: 33) were retrieved after screening 71 individuals; however, only 44 (NAT: 13; AAP: 31) proceeded to analysis due to profuse apical bleeding. The median patient age was 44 [29–55] (NAT: 63 [46–67]; AAP: 37 [28–51]) with an approximate 1:1 male‐to‐ female ratio. Three participants (NAT: 1; AAP: 2) were of Afro‐Caribbean descent, 7 were Asian (NAT: 2; AAP: 5) and 34 were Caucasian (NAT: 10; AAP: 24). Eighteen (NAT: 6; AAP: 12) samples were collected from mandibles and 26 (NAT: 7; AAP: 19) maxilla with incisors/canines being the commonest tooth (18; NAT: 2; AAP: 16) followed by molars (14; NAT: 4; AAP: 10) and premolars (12; NAT: 7; AAP: 5). Groups were matched by gender, ethnicity and inter‐arch tooth position’.

#### Item 6c: Results—The distribution of severity of disease in those with the target condition must be described, if possible

##### Explanation

The severity or stage of disease may also affect the test results, with severe cases often being more successfully identified. In diseases with a spectrum, for example, pulpitis, the accuracy of a test may depend on the proportions of study participants with severe, moderate or mild pulpitis, or using other qualifiers such as the depth of the caries lesion according to a bitewing radiograph. A clear description of the distribution of severity helps the reader judge if the study results are applicable to a given clinical population.

##### Example 6c.1

From Singh et al. (2021)—‘A final sample of 112 teeth in seventy‐four patients (forty‐two males and thirty‐two females) with a median age of 26 years (range: 18–57 years) was evaluated. Amongst them 65 teeth had been previously root filled and 47 teeth had primary infections which underwent root canal treatment before surgery. Out of 112 teeth, 110 teeth had periapical pathoses, 45 teeth with apico‐marginal bone defects, 26 teeth with through and through periapical bone defects, 26 teeth with root resorptive defects, 19 teeth had intact buccal cortical plates, 55 teeth had dehiscence and 29 had fenestrations of buccal alveolar bone and 8 teeth had combined alveolar defects, 12 teeth with root perforations and 4 teeth with root fractures (2 VRF, 1 horizontal fracture in the apical third of root, 1 oblique fracture)’.

#### Item 6d: Results—The distribution of alternative diagnoses in those without the target condition must be described

##### Explanation

The characteristics of participants without the target condition also affect test accuracy. Completely healthy participants in the group without the target condition often increase the specificity of the index test, whilst participants with other conditions or diseases with similar symptoms as the target condition may render false positive results, reducing the specificity. The type, spectrum and frequency of alternative diagnoses must be described, if this information is available, or the lack of information made transparent. Whether the reference standard was obtained for healthy subjects or those with alternative diagnoses should also be mentioned.

##### Example 6d.1

From Daline et al. (2024)—‘Figure shows the flow of participants’, see Figure 1.

#### Item 6e: Results—The time interval and any clinical interventions between index test and reference standard must be described

##### Explanation

Clear descriptions of the timing and sequence of index test and reference standard, and any interventions in the interval between, must be provided. The ideal situation for the assessment of test accuracy would be that the index test and reference standard are evaluated at the same time, but in many clinical situations this may not be possible. If the time interval between the index test and reference standard measurements is prolonged, the index test accuracy may be compromised by changes (worsening or improvement) in the target disease or in alternative conditions. The same compromise may occur if clinical interventions are performed between index tests and reference standard assessments. If the delay is systematically different between those with a positive test result and those with a negative result or between participants with different clinical characteristics (such as with and without symptoms) it may also bias the test accuracy.

##### Example 6e.1

From Mendoza et al. (2021)—‘Pregnancy‐associated plasma protein‐A(PAPP‐A) and placental growth factor (PlGF) were assessed before 11 weeks in 1675 (63.4%) of the 2641 women, and at or after 11 weeks in 966 (36.6%). Ninety (3.4%) women developed Pre‐eclampsia (PE), including 30 (1.1%) cases of preterm PE and 11 (0.4%) of early‐onset PE. Five (45.5%) cases of early‐onset and 16 (53.3%) of preterm PE were identified in the group in which serum biomarkers were assessed at 8 + 0–10 + 6 weeks and six (54.5%) cases of early‐onset and 14 (46.7%) of preterm PE in the group in which serum biomarkers were assessed at 11 + 0–13 + 6 weeks.

In the prediction of early‐onset PE and preterm PE using the Gaussian algorithm, no significant differences were observed in the areas under the ROC curves (AUCs) for any of the combinations of markers evaluated when the biochemical markers were assessed at 8 + 0–10 + 6 weeks compared with 11 + 0–13 + 6 weeks’.

##### Example 6e.2

From Dastmalchi et al. (2012)—‘After performing all pulp tests for the selected tooth, root canal treatment was performed in the following days. After completion of the access cavity, the vitality status of the teeth was recorded by a direct visual inspection, which was considered to be the gold standard in this study. If there was any doubt about the vitality status of the tooth, that tooth was excluded from the study’.

#### Item 6f: Results—A cross‐tabulation of the index test results (or their distribution) by the results of the reference standard must be provided

##### Explanation

The results should be verifiable by other researchers or readers. The data to allow this must be provided by the authors. A table presenting the numbers of participants with positive and negative index test results against reference standard results allows the reader to recalculate the disease prevalence and the reported measures of accuracy as well as any additional measures, for example, predictive values or perform a meta‐analysis.

##### Example 6f.1

From Kruse et al. (2019)—‘Table shows the cross‐tabulation of the results’, see Table 1.

**TABLE 1:** Item 6f – Cross table of the results. Reprinted from International Endodontic Journal, Vol 52, Kruse, C., Spin‐Neto, R., Evar, Kraft, D.C., Vaeth, M., Kirkevang, L.L. (2019) Diagnostic accuracy of cone beam computed tomography used for assessment of apical periodontitis: An ex vivo histopathological study on human cadavers. Reprinted from *International Endodontic Journal* 52, Pages No. 439–450, Copyright (2019) with permission from Wiley.

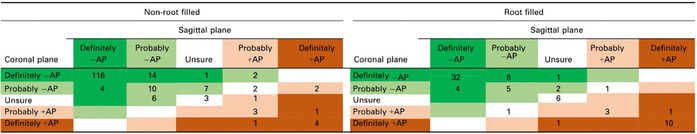

*Note*: Colouring of the individual entries indicates the combined CBCT‐based assessment.

Abbreviations: AP, apical periodontitis; CBCT, Cone Beam Computed Tomography.

#### Item 6g: Results—Estimates of diagnostic accuracy and their precision must be provided

##### Explanation

The precision of the point estimates of accuracy, usually reported as the 95% confidence intervals, shows the interval in which the ‘true’ sensitivity and specificity are expected to be. A study with a small number of participants usually has lower precision than a large study and providing a measure of the precision of each measure helps to avoid over‐optimism about the test accuracy in such cases.

##### Example 6g.1

From Kruse et al. (2019)—‘Table shows the diagnostic accuracy estimates’, see Table 2.

**TABLE 2:** Item 6g – Diagnostic accuracy estimates. Reprinted from International Endodontic Journal, Vol 52, Kruse, C., Spin‐Neto, R., Evar, Kraft, D.C., Vaeth, M., Kirkevang, L.L. (2019) Diagnostic accuracy of cone beam computed tomography used for assessment of apical periodontitis: An ex vivo histopathological study on human cadavers. *International Endodontic Journal* 52, Pages No. 439–450, Copyright (2019) with permission from Wiley.

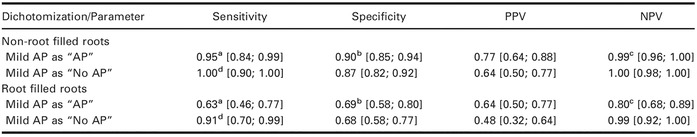

*Note*: The superscript letters indicate significant differences between nonroot filled and root filled roots (*P* < 0.001).

Abbreviations: AP, apical periodontitis; CI, confidence interval; NPV, negative predictive value; PPV, positive predictive value.

#### Item 6h: Results—Any adverse events from performing the index test or the reference standard must be described

##### Explanation

Reporting adverse events provides information on the probability of complications and the safety of patients when applying the index test and reference standard, which in addition to the test accuracy are factors that the clinician must consider when selecting a test for application in a clinical situation. The number (frequency) and character of any adverse events must be described for guidance.

##### Example 6h.1

From Hamamoto et al. (2023) – ‘Out of the 301 participants, adverse events were experienced by 8 individuals. These adverse events included three cases of bleeding, three cases of pancreatitis, one case of infection, and one case of pain. Within this group, one participant (3.1%) belonged to the hypervascular lesion group, while seven participants (2.6%) were in the hypovascular lesion group. Importantly, there were no statistically significant differences observed between these two groups in terms of the occurrence of adverse events’.

#### Item 6i: Results—Any further analyses (if applicable), including subgroup analyses and adjusted analyses, must be described, with a distinction made between pre‐specified and exploratory analyses

##### Explanation

The main results pertaining to the study objectives and/or hypotheses are sometimes supplemented by additional analyses focusing on specific questions. The results of any further analyses stated in the Methods must be described. In line with item 5y, a distinction must be made between pre‐specified and post‐hoc (exploratory) subgroup analyses since the latter may induce a higher risk of bias.

##### Example 6i.1

From Dianat et al. (2021)—‘Subgroup analyses revealed that the distance of >5 mm from buccal cortical plate was significantly associated with lower accuracy, increased operation time and greater incidence of mishaps in the FH group (*p* < .05), but not in the DNS group’.

#### Item 7a: Discussion—The key results must be summarized with reference to the study aim(s) and objective(s)

##### Explanation

The key results of the study must be summarized in relation to the study aim(s) and/or objective(s) to safeguard the relevance of the reported results for the research question and to help readers understand the main takeaway points of interest. This discussion also includes consideration of what the study has added to previously published knowledge and a comparison of the results with existing knowledge.

##### Example 7a.1

From Singh et al. (2021)—‘CBCT detected 9% (10 teeth) additional periapical radiolucencies when compared to PR. Of these, 80% were anterior and 20% posterior teeth (7 maxillary incisors, 1 mandibular incisor, 1 maxillary premolar, 1 maxillary molar). Surprisingly, 3 teeth with lesions larger than 4.5 mm and perforating the buccal cortex went undetected on PR (Table 2). These results concur with those of previous investigations with similar sample size (with reported additional detection rates of periapical lesions on CBCT image being 13.2%, 10.4%, 28%, 19.7%, 21% and 20%, respectively). Similarly, Uraba et al., concluded that the ability of CBCT imaging to detect periapical lesions that are not detectable on PR was significantly higher in maxillary incisors/canines. The sensitivity of PR and CBCT for detection of periapical lesion in the present study was 91% and 100% and the difference in sensitivity of both were significant. Lack of a well‐defined border of the lesions and anatomical noise from palatal alveolar bone might have led to the false negative diagnoses made from the periapical radiograph’.

##### Example 7b.2

From El Sayed and Gaballah (2021)—‘The results of the current study are consistent with previous reports that showed great difficulty in accomplishing the profound pulpal anesthesia for mandibular molar with irreversible pulpitis. The failure rate of IANB in the present study was 57% based on the gold‐standard test. The pulpal anesthetic failure may be due to the presence of pulpal inflammation, abnormal neural anatomy, and patients' anxiety’.

#### Item 7b: Discussion—Implications for practice must be described, including the intended use and clinical role of the index test

##### Explanation

Diagnostic testing has several purposes and possible applications, such as screening, prediction, treatment selection, follow‐up and prognosis. Depending on the intended clinical application of the test, the requirements of diagnostic accuracy of a test may differ, for example: in a screening test applied to rule out disease, high sensitivity may be required, whilst for a test applied as follow‐up after treatment (rule in disease) the clinician may place more importance on high specificity (Pewsner et al., 2004). The reported accuracy in relation to the clinical role of the test should be discussed and authors should elaborate on whether the accuracy estimates are sufficient, in other words, how well the index test is suited for the intended purpose. If the index test was found to be suited, its proposed position in the clinical pathway should also be discussed, with a comparison to other tests with a similar purpose if such exists.

##### Example 7b.1

From Hazard et al. (2021)—‘The present study assessed the accuracy of cold sensibility testing in patients with FCR [*full‐ coverage restorations*]. It is important for clinicians to appreciate that this test forms an important part of the clinical assessment process and should not be considered inappropriate. Although it was found that FCR teeth had lower accuracy to cold sensibility testing when compared to those with NC, the overall sensitivity was still high (86.6%). This was particularly the case for teeth with a pulpal diagnosis of symptomatic irreversible pulpitis’.

#### Item 7c: Discussion—The strength(s) of the study must be indicated

##### Explanation

The Discussion should describe the unique benefits of the study and how its findings add to the knowledge base in endodontics. The benefit for patients and/or other improvement in clinical practice must be described but not over‐emphasized or exaggerated.

##### Example 7c.1

From Virdee et al. (2023)—‘The Target‐48 panel was selected due to several advantages over other arrays. Only 1 μL of sample was required to simultaneously quantify 45 cytokines, the proximity extension technology offered exceptionally high sensitivity and specificity and prior *q*‐values demonstrate low false positive rates of analyte detection. Furthermore, pilot investigations confirmed that the eluting buffer or exudate matrix did not interfere with internal assay controls, as was observed with other immunoassays’.

#### Item 7d: Discussion—Study limitations must be indicated, including sources of potential bias and statistical uncertainty. Efforts to address bias must also be discussed

##### Explanation

All studies have limitations, and not all sources of bias can be avoided. Some important issues to consider and discuss are potential sources of bias, the precision of accuracy estimates and the applicability to clinical practice. Authors must reflect on weak points where flaws or deficiencies in, for example, study design, study setting, participant selection, selection or usage of reference standards or procedures may potentially have influenced the results. Any measures taken to avoid bias must also be described and their effectivity estimated.

##### Example 7d.1

From Virdee et al. (2023)—‘The present results nevertheless, still need to be interpreted with caution due to several methodological limitations. Most notably, it was not possible to normalize crude analyte values to TPC due to low yields found in NATs, an issue similarly observed by Zehnder et al. in dentinal tubular fluid. Normalizing to TFV, an alternative strategy utilized in several prior investigations and suggested by Zehnder and Belibasakis may thus be necessary when comparing transudates to exudates. This approach, however, increases risk of type 1 errors as differences in analyte concentration may be overestimated, which is why initial alpha values were set to 0.01’.

#### Item 7e: Discussion—The discussion of the strengths and weaknesses should be summarized in an overall assessment of the internal validity of the study

##### Explanation

To help readers assess the internal validity, the authors must summarize strengths and weaknesses and conclude which level of confidence can be placed in the study results, that is, how well the study succeeded in answering the research question.

##### Example 7e.1

From Singh et al. (2021)—‘The likelihood of type I (false positive) error, identifying presence of fenestration in CBCT when there were none surgically was 10%. Because of the limited spatial resolution of CBCT, bone thickness less than 0.6 mm are more likely to go undetected on the tomographic images. This might have accounted for the false positive findings.

In the present study, the patient's clinical data were made available to the observers interpreting the results of the diagnostic imaging, which mimicked a real‐life scenario where a clinician makes a decision based on both. This increased the sensitivity of PR with less effect on specificity. The sensitivity and specificity of CBCT, however, did not change even when the clinical data was made available. This should be considered in the future research as the performance of the test undertaken might be affected by the availability or absence of the patient's clinical information’.

#### Item 7f: Discussion—The generalizability (external validity, applicability, ‘real‐world’ relevance) of the study findings must be discussed

##### Explanation

To help the reader assess the relevance and applicability of the study results in different clinical, geographical or other contexts, the direction and magnitude of potential bias must be discussed with a focus on how it may affect the generalizability of the results.

##### Example 7f.1

From Schloss et al. (2017)—‘The increased radiation exposure of CBCT imaging limited the available sample size compared with the overall number of patients who had received surgery between 2011 and 2013. Thus, the overall distribution of successful versus failed cases may not be representative for the overall population, and as a result the dichotomized ratio of successful versus failed cases was not reported as an outcome rate. The primary reasons for CBCT acquisition were assessment of inconclusive healing and evaluation of odontogenic versus nonodontogenic symptoms. Although possibly not representative for the overall population, this subpopulation allowed for the investigation of the 2 central hypotheses (ie, that there were no differences in outcome classifications based on 2D periapical films versus 3D CBCT images and that inconclusive healing assessment would not be different between 2D and 3D imaging)’.

#### Item 7g: Discussion—Based on limitations in internal and external validity, implications for future research may be indicated when relevant

##### Explanation

The limitations of the study and their potential effects on its validity may have revealed areas of improvement, for example, in study design, selection of participants or procedure. The implications for future similar research should be discussed, and future directions should be suggested with a focus on the improvement of the identified deficiencies, when relevant.

##### Example 7g.1

From Singh et al. (2021)—‘CBCT had a sensitivity and positive predictive value (PPV) of 89.5% and negative predictive value (NPV) of 98% for predicting intact buccal cortical plate. This NPV value is higher than those reported by Mayo et al. (2019) suggesting strongly that when an intact buccal cortical plate was not identified in the CBCT image, it was less likely to be found after flap reflection. It should be noted that CBCT appears to be good positive predictor of dehiscence (PPV=95%). The increased PPV for dehiscence could be due to its increased prevalence of 56%. This is in contrast to the study of Mayo et al. who reported PPV of 31%—43% with low prevalence of only 7%. However, more clinical studies with accurate surgical bone measurements are needed in future’.

#### Item 8a: Conclusion—A rationale for the conclusion(s) must be provided

##### Explanation

The conclusion(s) must be aligned with the study aims, fully supported by the results and a balanced outcome of the discussion (e.g., considering potential bias). No conclusions must be made about issues that were not investigated in the study or were not in the aims and objectives of the study. Over‐generalization or extrapolation to other populations and settings not demonstrated in the results of the study must be avoided.

##### Example 8a.1

From Singh et al. (2021)—‘CBCT was associated with significantly greater diagnostic accuracy for detection of periapical lesion, apico‐marginal bone defects, through and through bone defects and root perforations compared to periapical radiographs. PR was equally effective as CBCT in identifying healthy periapical tissues. There was no significant difference between the imaging techniques in diagnosing external root resorptive defects and root fractures. CBCT failed to diagnose apico‐marginal bone defects in 33% teeth. When evaluating the status of buccal cortical plate from CBCT images, observers could detect the absence of bone better than its presence as implied from the high sensitivity values for fenestration and dehiscence. Limited FOV CBCT should be considered for selective cases where periapical radiography exhibits diagnostic ambiguity’.

#### Item 8b: Conclusion—The conclusion(s) must be stated explicitly and address all the study aims and objectives

##### Explanation

The conclusion(s) must be identified as such, and clearly stated. All study objectives must be addressed. If the results were inconclusive for any of the objectives, this must be described.

##### Example 8b.1

From Zhang et al. (2019)—‘The diagnostic accuracy of CBCT imaging for VRFs remains controversial. Based solely on a fracture line, sensitivity and accuracy was poor in the diagnosis of subtle VRFs in endodontically treated teeth in this study. However, vertical bucco‐palatal (lingual) bone loss was found in most VRF teeth and therefore could be an important indirect sign for the diagnosis of VRFs. Furthermore, this type of bone loss could be indicated on CBCT images’.

#### Item 9a: Source of funding—The sources of funding and other support (such as donation of drugs, instruments and equipment) and the role of the funder(s), (such as whether they approved, consulted, co‐authored or contributed to the manuscript prior to submission) in the study must be acknowledged and described

##### Explanation

Information about the funding source of the study and the role of the funder(s) allows the reader to judge if there is any potential conflict of interest or risk of bias associated with sponsorship of the study. Sponsorship in the form of funding does not bar the study from publication but must be transparent for the readers to appreciate the risk of bias.

To preserve the double‐masked peer‐review process, details such as grant numbers or specific university information should not be given at the stage of submission but must be provided before publication once the peer review has been completed.

##### Example 9a.1

From Virdee et al. (2023)—‘This project has been supported by the following grants: (1) 2022 Glaxo‐Smith‐Kline Grant: Oral & Dental Research Trust; (2) 2020 Young Investigator Grant: European Society of Endodontology; (3) 2017 Annual Research Grant: British Endodontic Society. The first author was awarded the 2023 National Poster Prize at the British Endodontic Society Spring Scientific Meeting for the work presented in this manuscript’.

#### Item 10a: Conflict of interest—An explicit statement on conflicts of interest must be provided, together with full affiliations of the authors

##### Explanation

A conflict of interest between the researcher(s) and an external agent could be professional, legal, commercial, or financial in nature. The authors must provide full affiliations, and any relationships with the potential to influence the outcome of the study (induce bias) must be explicitly disclosed as a conflict of interest. Examples of such relationships are ownership of stock or patent in a company, company membership, committee or advisory board membership, and acting as a consultant for or receiving speaker's fees from a company associated with the study. Such conflicts of interest do not bar the study from publication but must be transparent for readers to appreciate the risk of bias. If there are no conflicts of interest this should be declared.

##### Example 10a.1

From Virdee et al. (2023)—‘The authors deny any conflicts of interest related to this study’.

##### Example 10a.2

From Hinckfuss et al. (2023)—‘Declaration of competing interest. The authors declare the following financial interests/personal relationships which may be considered as potential competing interests: R. G. Loeb has received $1000 per year to be on the Masimo Inc. Scientific Advisory Board. P. M. Sanderson is co‐inventor of a respiratory sonification (Sanderson and Watson, US Patent 7070570). No other authors have competing interests to declare’.

#### Item 11a: Quality of images—The text or caption must include information about the equipment, software and settings used to create all image(s)

##### Explanation

Details of the equipment (model/version, supplier, city and country) and methods used for image acquisition and software (version, supplier city and country) used for analysis must be provided. The make, manufacturer, model/version number and settings (e.g., kV, mA, voxel size, resolution, FoV, weighing in MRI, etc.) of the equipment used to collect the data (capture, record or reproduce images) must be provided, along with the software developer and specific versions used for image processing. If the images were analysed statistically, the name, details of the developer and version of the statistical package software must be provided. Any modification of the image(s) must be described.

##### Example 11.a.1

From Zhang et al. (2019)—‘CBCT images were acquired using a NewTom VG scanner (QR srl, Verona, Italy) according to the manufacturer's instructions with a voxel size of 0.125 mm. CBCT images were analyzed using the built‐in software NNT 5.3 (J Morita Manufacturing Corp, Kyoto, Japan) using a 29.7‐inch RadiForce MX300 W (Eizo Nanao Corporation, Hakusan, Japan) screen with a resolution of 2560 × 1600 pixels. Coronal, sagittal, and axial planes at different root levels were displayed on the monitor’.

#### Item 11b: Quality of images—The purpose for acquiring the image(s) and the reasons for including it/them in the publication must be explained in the text

##### Explanation

Use of every image in a publication must be justified, for example by a need to illustrate the diagnostic procedures, for example, the index test or reference standard. All images should have the highest possible resolution and be of adequate quality for their purpose to be fulfilled. Descriptive information should accompany the image to ensure the reader's understanding of what the image is meant to illustrate and any possible limitations.

##### Example 11b.1

From Davies et al. (2016)—‘Figure 4 – The 26 was still symptomatic 1 year after treatment. The periapical shows a reduction in the periapical area around the mesial root, but the CBCT reconstructed images show the area to have increased (blue arrows). In addition, when assessing the CBCT reconstructed images, it is possible to identify an unfilled MB2 canal (red arrow) which could be the cause of failure. Retreatment would therefore be the most suitable management option in this case’.

#### Item 11c: Quality of images—The authors must provide the circumstances (conditions) under which the image(s) were viewed and appraised in the text

##### Explanation

Assessment and interpretation of images is a process that may be influenced by several factors. For example, the examiner(s) credentials/training and any calibration undertaken to improve the interpretation performance should be described. Another factor is image quality. Image equipment (such as image resolution, size, pixel ratio, etc.) and (if applicable) additional tools used by the examiners when interpreting the images should be presented.

In cases when more than one examiner has interpreted the images, or if one examiner interpreted the images more than once, the level of agreement between assessments should be given as inter‐ or intra‐examiner agreement (kappa statistics, intraclass correlation coefficient [ICC], proportion of concordant evaluations or other relevant measure of agreement or statistical analysis).

##### Example 11c.1

From Singh et al. (2021)—‘Radiographic images were evaluated concurrently by three blinded observers (an oral and maxillofacial radiologist [AG] and two endodontists [RY, JD]) well experienced in radiographic analysis. One case from each category was utilized for calibration preceding the image analysis which were subsequently excluded from the study (nine teeth from 7 patients). In first session, the clinical history and examination along with periapical radiographs were analysed by each observer individually. At the second evaluation, in addition to clinical data and periapical radiographic findings, the same observers were provided with CBCT images of the patients in a randomized order at the interval of at least 2 weeks. Disagreements were discussed to reach consensus.

Kappa analysis revealed the inter‐observer reliability to 0.79 for PR and 0.83 for CBCT and intra‐observer reliability for PR and CBCT to be 0.85 and 0.92 for observer 1, 0.84 and 0.87 for observer 2 and 0.79 and 0.85 for observer 3 respectively’.

#### Item 11d: Quality of images—The image capture settings including resolution, magnification as well as any *post‐hoc* manipulation or enhancement (e.g., brightness, colour balance, smoothing and staining) must be specified in the text or legend

##### Explanation

Any editing or enhancement of the image(s) from its original resolution or magnification must be described. If relevant, a scale bar with measurements (e.g., histogram, WW/WL) should be provided to clearly show modifications in size and magnification. Any justified modifications or enhancements should be applied to the entire image and not limited to specific details, causing distortion of the image, which could potentially lead to misinterpretation by the readers. Undisclosed image modifications, particularly if they appear to conceal, incorrectly portray or falsify data, are unacceptable and may constitute scientific misconduct (Lang et al., 2012; Rossner & Yamada, 2004).

##### Example 11d.1

From Kruse et al. (2017)—‘All PR images and CBCT volumes were viewed in a quiet room with subdued lighting using two 24‐inch flat screen monitors, Dell P2412H (Dell Inc., Round Rock, TX); one monitor to display the baseline PR (the immediate post‐operative periapical image taken after the initial SER) and the other monitor for the 7‐year follow‐up PR or CBCT. Dedicated PACS software (DigiView, developed by programmer Erik Gotfredsen, Section of Oral Radiology, Aarhus University, Aarhus, Denmark) was used to display the periapical radiographs, and the observers were able to enhance viewing parameters (*e.g*., brightness and contrast). Software for viewing 3D image volumes (OnDemand, Cybermed Inc., Daejeon, South Korea) was used for a dynamic evaluation of the CBCT volumes, and the observers could freely change all visualization parameters and perform individual sectioning’.

#### Item 11e: Quality of images—An interpretation of the findings (meaning and implications) from the image(s) must be included

##### Explanation

All relevant information derived from the interpretation of images, including the meaning of the finding(s) regarding the study question and the clinical implications (if applicable), must be provided.

##### Example 11e.1

From Singh et al. (2021)—‘Through and through (T&T) defects (a) periapical radiography reveals focal radiolucency overlying existing radiolucency suggestive of T&T bone defects (white circle) (b)‐ (d) sagittal, coronal and axial CBCT views showing combined apico‐marginal and T&T bone defect in tooth 21 (e) after flap reflection showing false positive CBCT result in detection of apico‐marginal bone defect of tooth 21 (f) intraoperative presence of T&T bone defects in teeth 21 and 22’.

#### Item 11f: Quality of images—The legend must explain precisely what the subject is and what subject features it depicts. Images of patients should indicate their age, sex and, if applicable, ethnicity

##### Explanation

The legends for images should be simple to understand, complete and include pertinent demographic information. Details regarding the image views, for instance, the type of radiographic or CBCT view, should be provided. The legends should enable the reader to understand the message that the image portrays within the context of the study.

##### Example 11f.1

From Zhang et al. (2019)—‘Figure 1. An example of a tooth with a subtle incomplete fracture. (A) A PR taken after clinical examination showed that RCT was performed well. Lateral radiolucent areas were observed at the mesial aspect of the tooth. (B) A coronal reconstruction CBCT image along the axis of the root showed that vertical palatal alveolar bone loss had reached the apical region. (C) The extracted tooth showed the fracture line on the palatal side of the root. (D) D1–D3 were axial images showing streak artefacts on the palatal side of the root. (E) A micro‐CT image showed a subtle incomplete fracture line on the palatal side of the root *in vitro* with a voxel size of 0.02 mm’.

#### Item 11g: Quality of images—Markers/labels must be used to identify the key information in the image(s) and defined in the legend

##### Explanation

The specific features or areas of importance of an image (explaining its inclusion) should be identified and indicated using markers or labels, such as arrows. The markers/labels should be of adequate size and appropriate colour to be clearly observed but must not obscure other important features of the image that must be possible to assess to understand the image correctly (such as the root apex or marginal bone level). Markers or labels must be explained in the legend to help the reader understand what they are meant to emphasize (Lang et al., 2012). The image and legend should be self‐explanatory, that is: possible to understand without access to the corresponding text in the manuscript.

##### Example 11g.1

**FIGURE 2:**
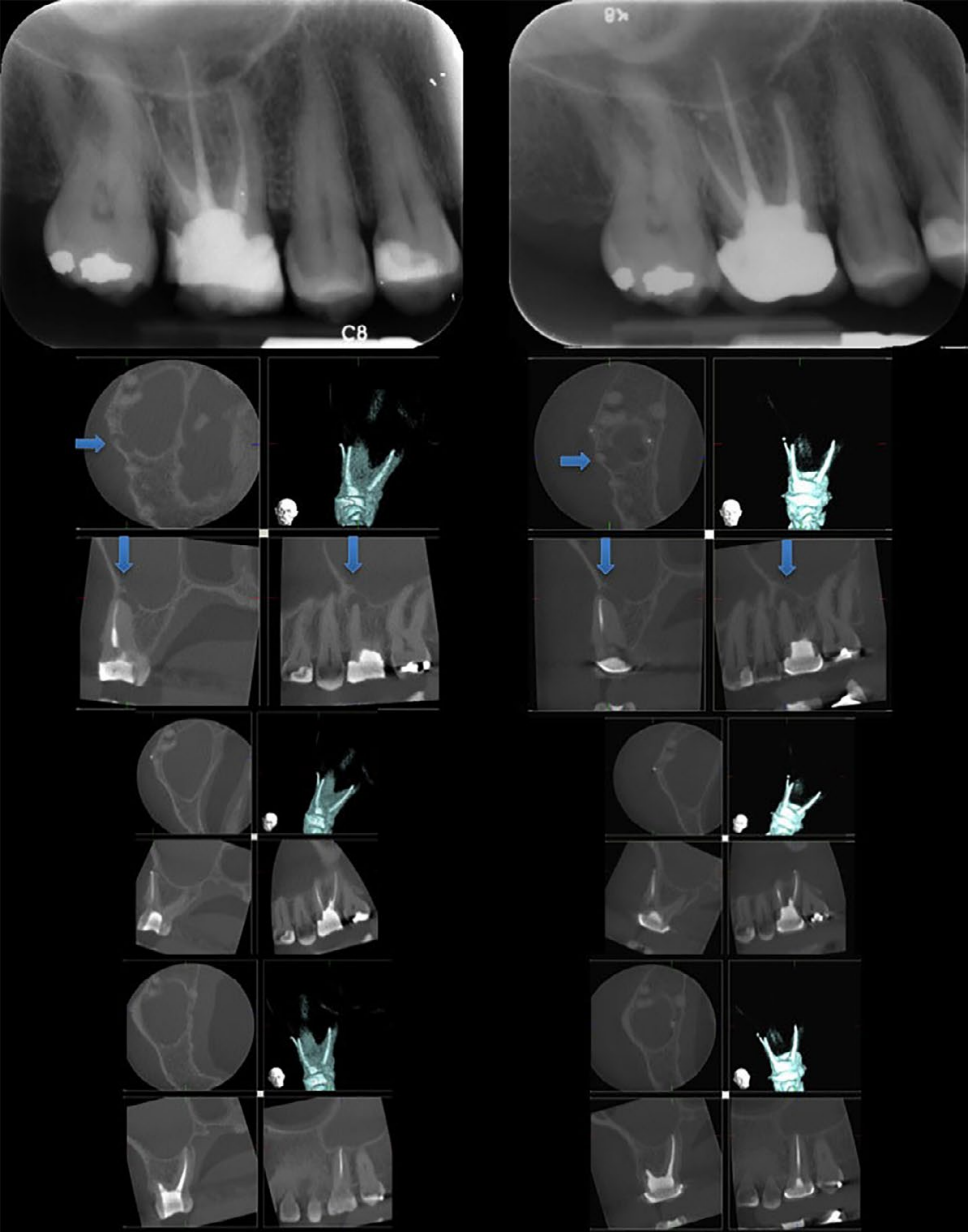
Item 11g – Example of a case detailing all the images made available for the examiners. They had the entire CBCT scan to view if they wished. (a, b) Preoperative and 1 year postoperative periapicals of the 16 showed apparent reduction of the periapical lesion associated with the mesial root. (c, d) Preoperative and 1 year postoperative reconstructed images of the 16 mesial roots in axial, coronal and sagittal planes showing enlargement of the periapical lesion associated with the root (arrowed). (e,f) Preoperative and 1 year postoperative reconstructed images of the distal root showing maintenance of a healthy periapex. (g, h) Preoperative and 1 year postoperative reconstructed images of the palatal root showing maintenance of a healthy periapex. Reprinted from *International Endodontic Journal*, Vol 49, Davies, A., Patel, S., Foschi, F., Andiappan, M., Mitchell, P. J., & Mannocci, F. (2016). The detection of periapical pathoses using digital periapical radiography and cone beam computed tomography in endodontically retreated teeth – part 2: A 1‐year post‐treatment follow‐up, pages No. 623–635, Copyright (2016) with permission from Wiley.

From Davies et al. (2016)—Arrows have been used to identify the key information and are mentioned in the legend, see Figure 2.

#### Item 11h: Quality of images—Patient(s) identifiers (names and patient numbers) must be removed, and all images must be anonymized or deidentified

##### Explanation

All personal identifying information, including name, patient identification number, eyes and so on, must be removed or obscured from all images, with special attention paid to any visual or verbal information that could lead to the individual's identification. This is in compliance with, for example, the European General Data Protection Regulation (GDPR; https://eur‐lex.europa.eu/eli/reg/2016/679/oj) and the Health Information Portability and Accountability Act (HIPAA; https://www.hhs.gov/hipaa/index.html).

##### Example 11h.1

**FIGURE 3:**
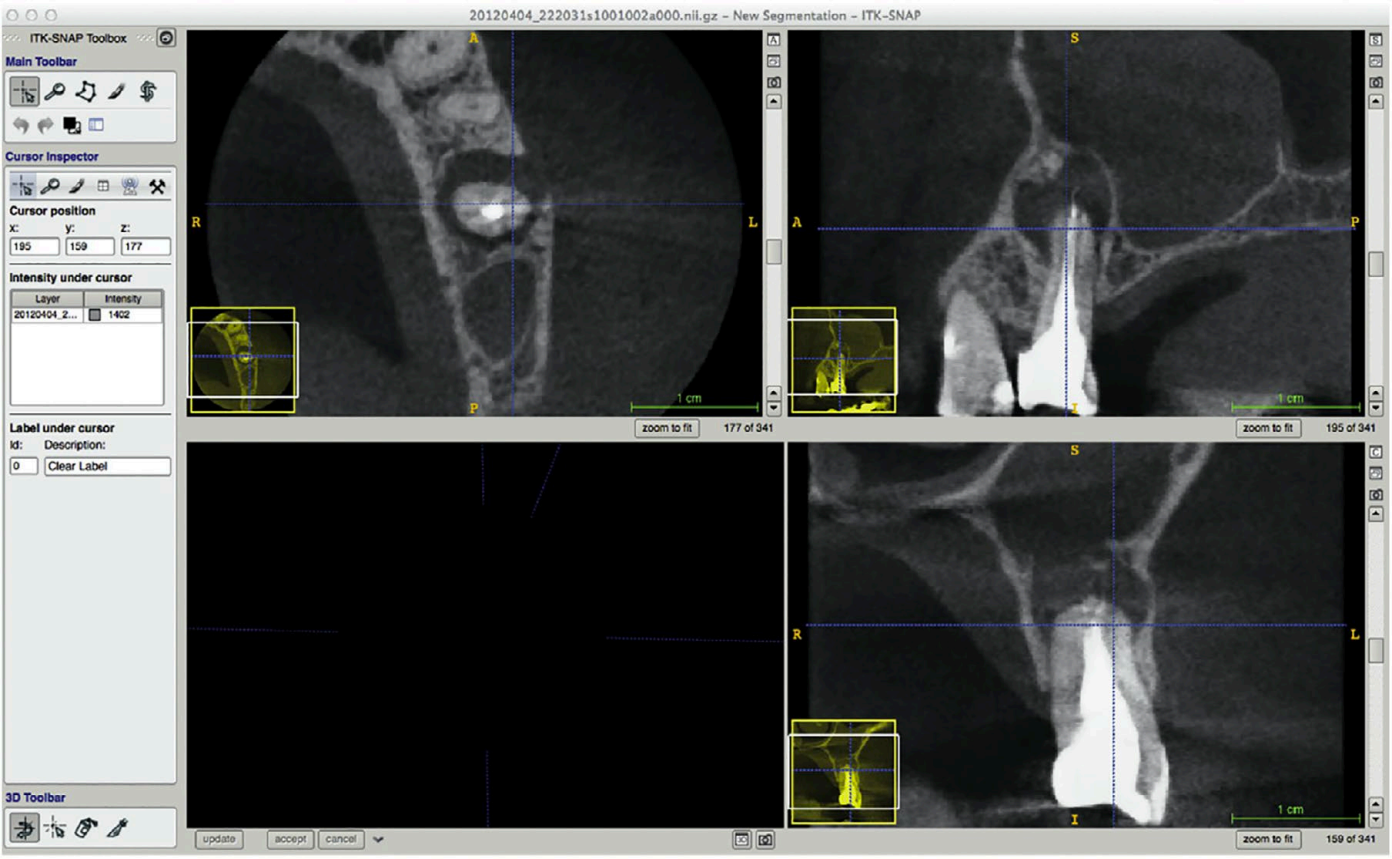
Item 11h – Deidentified CBCT images. Reprinted from *Journal of Endodontics*, Vol 43, Schloss, T., Sonntag, D., Kohli, M. R., & Setzer, F. C. A Comparison of 2‐ and 3‐dimensional Healing Assessment after Endodontic Surgery Using Cone‐beam Computed Tomographic Volumes or Periapical Radiographs, Copyright (2017) with permission from Elsevier.

From Schloss et al. (2017)—Deidentified CBCT images, see Figure 3.

#### Item 11i: Quality of images—If treatment was carried out, the legend of each image must state whether the image is pre‐treatment, intra‐treatment or post‐treatment, as well as how photographs and/or radiographs were standardized over time

##### Explanation

Information must be provided on when an image was obtained in the course of the study. For example, images could represent the index test, the reference standard, or both, and this must be described. The timing of each image (e.g., pre‐treatment, intra‐treatment, post‐treatment or at follow‐up) must be described and if multiple images are included, they should be presented and labelled in a chronological sequence based on the timing of obtaining the images or based on another rationale, which should then be explained.

##### Example 11i.1

From Davies et al. (2016)—‘Example of a case detailing all the images made available for the examiners. They had the entire CBCT scan to view if they wished. (a, b) Preoperative and 1 year postoperative periapicals of the 16 showing apparent reduction of the periapical lesion associated with the mesial root. (c, d) Preoperative and 1 year postoperative reconstructed images of the 16 mesial root in axial, coronal and sagittal planes showing enlargement of the periapical lesion associated with the root (arrowed). (e, f) Preoperative and 1 year postoperative reconstructed images of the distal root showing maintenance of a healthy periapex. (g, h) Preoperative and 1 year postoperative reconstructed images of the palatal root showing maintenance of a healthy periapex’.

## DISCUSSION

If a diagnostic test has poor validity in identifying health and disease, the diagnosis is at best delayed, and at worst incorrect and followed by ill‐advised interventions. Studies on diagnostic accuracy thus have an important role in endodontics but are challenging since the state of the affected pulp and periapical tissues is not easily identified. The true state of the tissues along the continuum of health and disease should preferably be determined before any intervention is undertaken, but if this is not possible a sound reference standard is not available. Histologic analysis of dental and surrounding tissues cannot be performed without invasive and irreversible procedures and is thus only feasible in cadaver studies or when pulp is extirpated in a patient, which limits its usefulness as a reference standard.

Clinically, the available information currently used to make an endodontic diagnosis includes patient‐reported symptoms and history, clinical and radiographic signs, and signs of altered nerve function such as lack of response or hyperreactivity of the pulpal afferents. Sometimes, the result of this ‘best available’ diagnostic procedure (such as the diagnostic entity *symptomatic irreversible pulpitis*) is used as the clinical reference standard in research studies. This obviously presents a two‐fold challenge: First, a circularity problem may arise (given that many features that index tests propose to measure are simultaneously part of the reference standard) and bias the validation of many potential clinical tests. Secondly, the accuracy of the reference standard itself may conceivably be uncertain because the diagnostic criteria themselves are often neither operationalized nor validated.

These issues, and others, make studies of diagnostic accuracy in endodontics challenging to design and undertake, which makes it even more important that robust protocols are reported rigorously. A systematic review of endodontic methods found that the majority of diagnostic studies had low quality (Swedish Council on Health Technology Assessment [SBU], 2010), which was partly attributed to a lack of information about important study characteristics. Many manuscripts submitted to endodontic journals are rejected due to deficient methodology and poor reporting quality (unpublished data, Paul MH Dummer, Henry F Duncan). As a consequence, inadequate quality of reporting is a threat to appropriate endodontic care, since many studies on diagnostic accuracy fail to reach the level of evidence required to contribute to clinical guidelines. Enhancing reporting quality is thus an important step towards more targeted care.

The PRIDASE 2024 is based on the generic guidelines for diagnostic accuracy studies, the STARD 2015 guidelines (Bossuyt et al., 2015; Cohen et al., 2016), and adapted and modified to specifically support studies within endodontics. One such modification is the incorporation of the Clinical and Laboratory Images in Publication (CLIP) principles (Lang et al., 2012) in the PRIDASE checklist. Reference standards and index tests in studies on diagnostic accuracy in endodontics can involve imaging (e.g., radiography) as well as laboratory tests and histology. To enable correct interpretations and conclusions, the quality of included images should be high, and important information about image acquisition and characteristics should be transparent to the readers. Hence, in parallel with the PRILE 2021 guidelines for reporting laboratory studies, the PRIDASE 2024 guidelines have incorporated the CLIP principles (Lang et al., 2012).

Furthermore, the PRIDASE checklist is aligned with the other five published guidelines for reporting studies in endodontology under the Preferred Reporting Items for study Designs in Endodontology (PRIDE) umbrella initiative (Nagendrababu & Dummer, 2020). The items in the PRIDASE 2024 checklist are listed according to the following sections: title, keywords, abstract, introduction, methods, results, discussion, conclusion, sources of funding, conflicts of interest and quality of images. However, authors are able to change the sequential order of the elements to ensure that every essential item is included in the manuscript in a structured and logical manner, thereby improving the reader experience.

This PRIDASE Explanation & Elaboration document supports the PRIDASE 2024 guidelines by helping authors understand the items in the checklist, and their importance in helping readers evaluate the usefulness of the study in their own context. The examples provided from published studies highlight how critical information may be presented clearly and succinctly. Furthermore, this explanatory document provides authors with a structure and additional support for developing a manuscript that includes all necessary information for the paper to be considered as high quality in a systematic assessment of evidence for the efficacy of the diagnostic method. To demonstrate this, manuscripts on diagnostic accuracy studies that follow the PRIDASE 2024 guidelines should include the following statement early in the Methods section ‘This study on diagnostic accuracy is reported according to the PRIDASE 2024 guidelines (Nagendrababu et al., 2024)’ with reference to the PRIDASE 2024 consensus publication (Nagendrababu et al., 2024). This recommendation parallels previously published reporting guidelines for other study types, for example, the Preferred Reporting Items for RAndomized Trials in Endodontics (PRIRATE) 2020 (Nagendrababu, Duncan, et al., 2020) and the Preferred Reporting items for OBservational studies in Endodontics (PROBE) 2023 (Nagendrababu et al., 2023).

Regardless of study type, a flowchart clearly and transparently displaying the key elements of the study is helpful to further improve the understanding of the study protocol (Cohen et al., 2016; Nagendrababu, Duncan, et al., 2020; Page et al., 2021). Manuscripts following the PRIDASE 2024 reporting guidelines are therefore expected to include a flowchart describing the flow of participants including the prevalence of the target condition in the study population as indicated by the reference standard, the index test results (negative, positive and inconclusive) and their relationship to the absence and presence of the target condition. Similarly, the inclusion of a flowchart is mandatory in the STARD 2015 guidelines (Bossuyt et al., 2015) as well as in most previously published endodontic‐specific guidelines (Nagendrababu & Dummer, 2020; Nagendrababu, Duncan, et al., 2020; Nagendrababu, Kishen, et al., 2021; Nagendrababu, Murray, et al., 2021).

## CONCLUSION

This Explanation & Elaboration document was written to provide additional support to authors on how to use the PRIDASE 2024 checklist when reporting on diagnostic accuracy studies in endodontics. It is anticipated that when applying the PRIDASE 2024 guidelines the quality of manuscripts submitted to journals will improve, and thereby the quality of available evidence for the efficacy of diagnostic methods to correctly identify pulp and periapical conditions. Journals can support this favourable development by requiring that reporting on diagnostic studies follow the PRIDASE 2024 guidelines. The authors of this document encourage all journals that accept diagnostic accuracy studies in endodontics to adopt this requirement in their author guidelines for manuscript preparation.

## AUTHOR CONTRIBUTIONS

All the authors made substantial contributions to the manuscript. All the authors have read and approved the final version of the manuscript.

## FUNDING INFORMATION

None received.

## CONFLICT OF INTEREST STATEMENT

The authors have stated explicitly that there are no conflicts of interest in connection with this article.

## ETHICS STATEMENT

Ethical approval was obtained from the University of Sharjah, Sharjah, UAE (REC‐20‐11‐06‐01).

## Data Availability

Data sharing is not applicable – no new data are generated, or the article describes entirely theoretical research.
